# Systemic clearance and brain distribution of carbazole-based cyanine compounds as Alzheimer’s disease drug candidates

**DOI:** 10.1038/s41598-017-16635-4

**Published:** 2017-11-27

**Authors:** Wei Zhou, Xiaohui Hu, Kin Yip Tam

**Affiliations:** Faculty of Health Sciences, University of Macau, Macau, SAR P.R. China

## Abstract

SLM and SLOH, two analogues of carbazole-based cyanine compounds, have been shown to inhibit β-amyloid peptide aggregation *in vitro* and in Alzheimer’s disease model mice, which could be potentially developed into drugs for disease treatment. To pave the way for further pharmacokinetics-pharmacodynamics study, we set to investigate these compounds’ systemic clearance pathways and their brain exposure. We found that they generally exhibited relatively low plasma clearance which comprised of hepatic clearance and biliary clearance. Phase I oxidative metabolites for SLM and for SLOH upon microsomes incubation were identified, and the metabolism by CYP3A4 were found to be the major (>70%) hepatic clearance pathway, while the efflux by P-gp and BCRP located in the canalicular membrane of hepatocytes led to high biliary clearance. The permeation of SLM and SLOH through the brain endothelium was affected by the efflux transporters (P-gp and BCRP) and influx transporter (OATP2B1). The unbound interstitial fluid to plasma ratio (*K*_puu,brain_) was 8.10 for SLOH and 11.0 for SLM, which favored brain entry and were several folds higher than that in wild-type mice. Taken together, these carbazole compounds displayed low plasma clearance and high brain permeability, which entitle further development.

## Introduction

Alzheimer’s disease (AD) is a neurodegenerative disorder, which leads to an impairment of many cognitive functions, particularly the short-term memory^1^. Multiple lines of evidence from biochemical, genetic and animal studies supports the theory that β-amyloid (Aβ) peptides of 40 and 42 residues, formed by enzymatic cleavage of amyloid precursor protein (APP), play a crucial role in the AD pathogenesis, where the aggregation of monomeric Aβ peptides to insoluble plaque-associated amyloid fibrils, such as annular protofibrills, cluster-like fibrils, *etc*. via soluble oligomeric intermediates, eventually lead to the neuronal cells death^2–5^. Thus, development of a blood brain barrier (BBB) permeable agent, which inhibits the formation of the neurotoxic amyloid oligomers and fibrils by interfering with the aggregation of the Aβ in interstitial fluid (ISF), has been regarded as a rational strategy to tackle AD over the years^6^.

**Figure 1:**
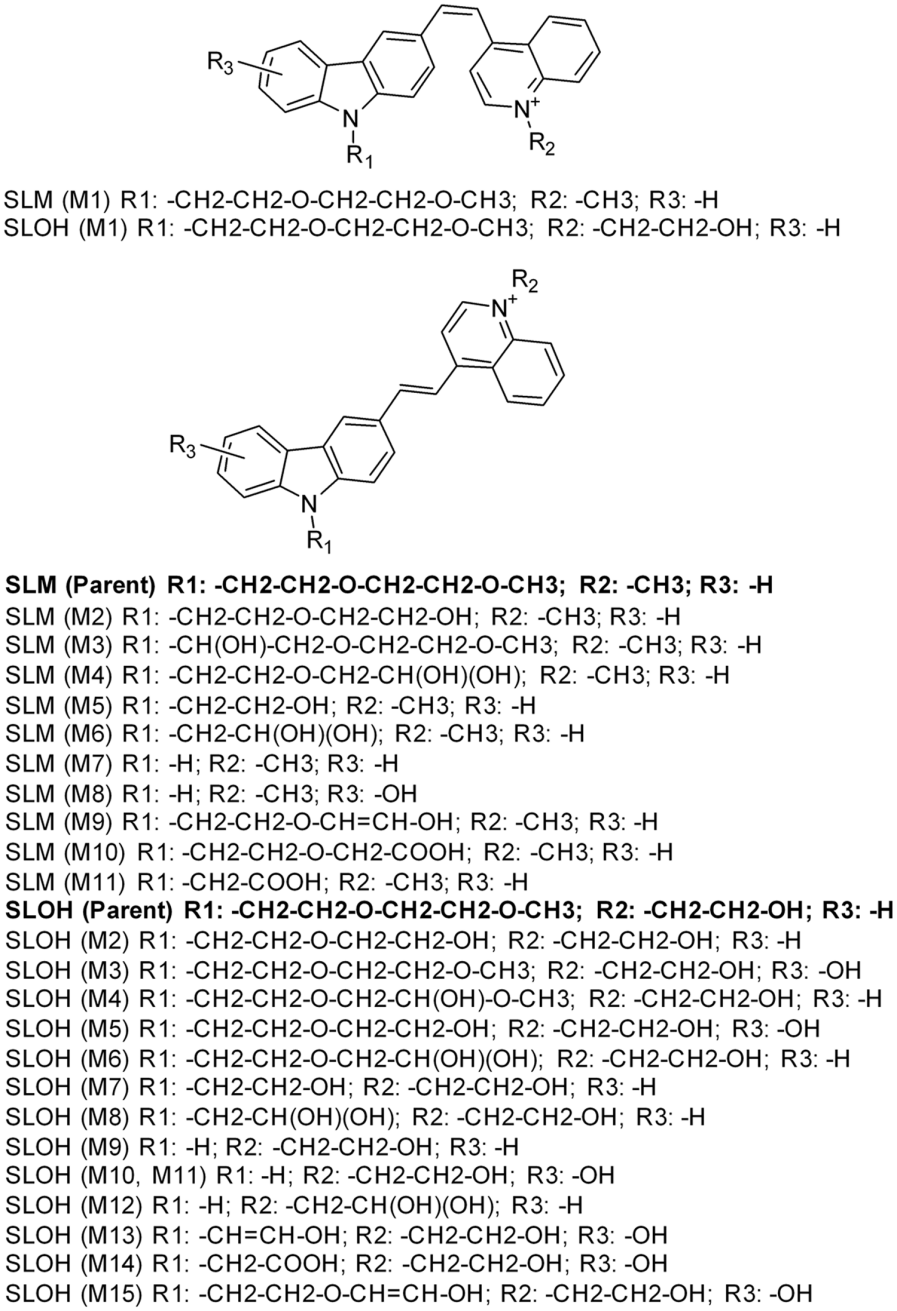
The chemical structures of SLOH and SLM and its microsome metabolites.

Recently, a series of carbazole-based cyanine compounds with various functionalized pyridinium or quinolinium moieties has been reported to inhibit the Aβ aggregation^7^. It was found that these inhibitors, namely (E)-4-(2-(9-(2-(2-methoxyethoxy) ethyl)-9H-carbazol-3-yl)-vinyl)-1-methyl-quinolin-1-iumiodide (SLM) and (E)-1-(2-hydroxyethyl)-4-(2-(9-(2-(2-metho-xyethoxy)-ethyl)-9H-carbazol-3-yl)vinyl) quinolin-1-ium chloride (SLOH) (Fig. 1)^8^, bound to Aβ_(1–40)_ with reasonably good affinity (μM), and were able to reduce Aβ fibrillogenesis significantly *in vitro*, and to alleviate the pathological symptom and memory deterioration in AD model mice^9^.

In order to pursue the next step for new compounds development, pharmacokinetics (PK)- pharmacodynamics (PD) study is necessary. PK usually describes the drug concentration-time courses in body fluids after administration. Acceptable PK properties are key in potential drug-like clinical candidate^10^. Whereas PD observes the magnitude of pharmacological response of therapeutic targets to drug under current concentration. Establishment of PK-PD models is to evaluate dose-concentration-response relationships and subsequently describe and predict the effect-time courses. The compound-target/system interactions, such as signal transduction, tolerance and slow receptor-binding, can be systemically studied by mechanistic processes, incorporated into PK/PD-modelling, which are essential in efficacy and safety prediction^11^. The expanded use of PK-PD models is thus highly beneficial for drug development as well as applied pharmacotherapy.

Prior to PK-PD study, it is important for us to study thoroughly the major clearance pathways of these compounds in the blood circulation and the brain exposure in ISF. This work elucidated the systemic clearance (hepatic, renal and biliary clearance) and brain penetration (transporters and exposure) mechanism of abovementioned carbazole-based cyanine compounds in liver microsomes, hepatocytes, Caco-2 cell, OATP1B1/1B3/2B1/1A2 stably transfected HEK293 cell and *in vivo* animal models.

## Results

### Blood cell partitioning, non-specific plasma protein binding, microsomes binding, hepatocytes binding, and brain distribution using brain slices

As shown in Table 1, the blood to plasma partition of SLM and SLOH was about 1.40 and 1.30 in mice and 0.820 and 0.760 in human, respectively, suggesting that SLM and SLOH were almost equally distributed into plasma and red blood cells. No significant reduction of SLM and SLOH were observed in plasma, microsomes and deactivated hepatocytes of all species for more than 4 hours at 37 °C (half-life elimination (*t*_1/2_) > 24 h). Besides, SLM and SLOH showed high plasma binding (>95%) in mice and human. *V*_u,brain_ values were all around 1.50 mL/g brain, well above 0.800 mL/g threshold, indicating possible intracellular distribution^12^.

**Table 1:** Blood cell partitioning, non-specific plasma, microsomes, hepatocytes and brain distribution by slices of SLM and SLOH (*n* = 3, Mean ± SD).

Parameters	SLOH		SLM	
	Mouse	Human	Mouse	Human
Blood to plasma ratio	1.30 ± 0.250	0.760 ± 0.0800	1.40 ± 0.100	0.820 ± 0.0800
*f*_u,plasma_ %	3.50 ± 0.700^$^	0.170 ± 0.0400	1.70 ± 0.380^$^	0.100 ± 0.0100
*f*_u,mic_ %	17.0 ± 2.40^$^	6.60 ± 1.60	11.0 ± 1.06^$^	7.30 ± 0.150
*f*_u,hep_, %	16.0 ± 5.50	5.50 ± 0.730^$$^	15.0 ± 4.60	11.0 ± 0.870^$$^
*V*_u,brain_ (WT) (mL/g brain)	1.40 ± 0.400	—	2.10 ± 0.650	—
*V*_u,brain_ (AD) (mL/g brain)	1.20 ± 0.240	—	1.50 ± 0.580	—

Statistical significance in parameters was estimated by student’s t-test. ^$^ *p* < 0.05 and ^$$^ *p* < 0.01, (SLOH *vs* SLM).

### Permeability and efflux transporters (P-gp and BCRP) identification

**Figure 2:**
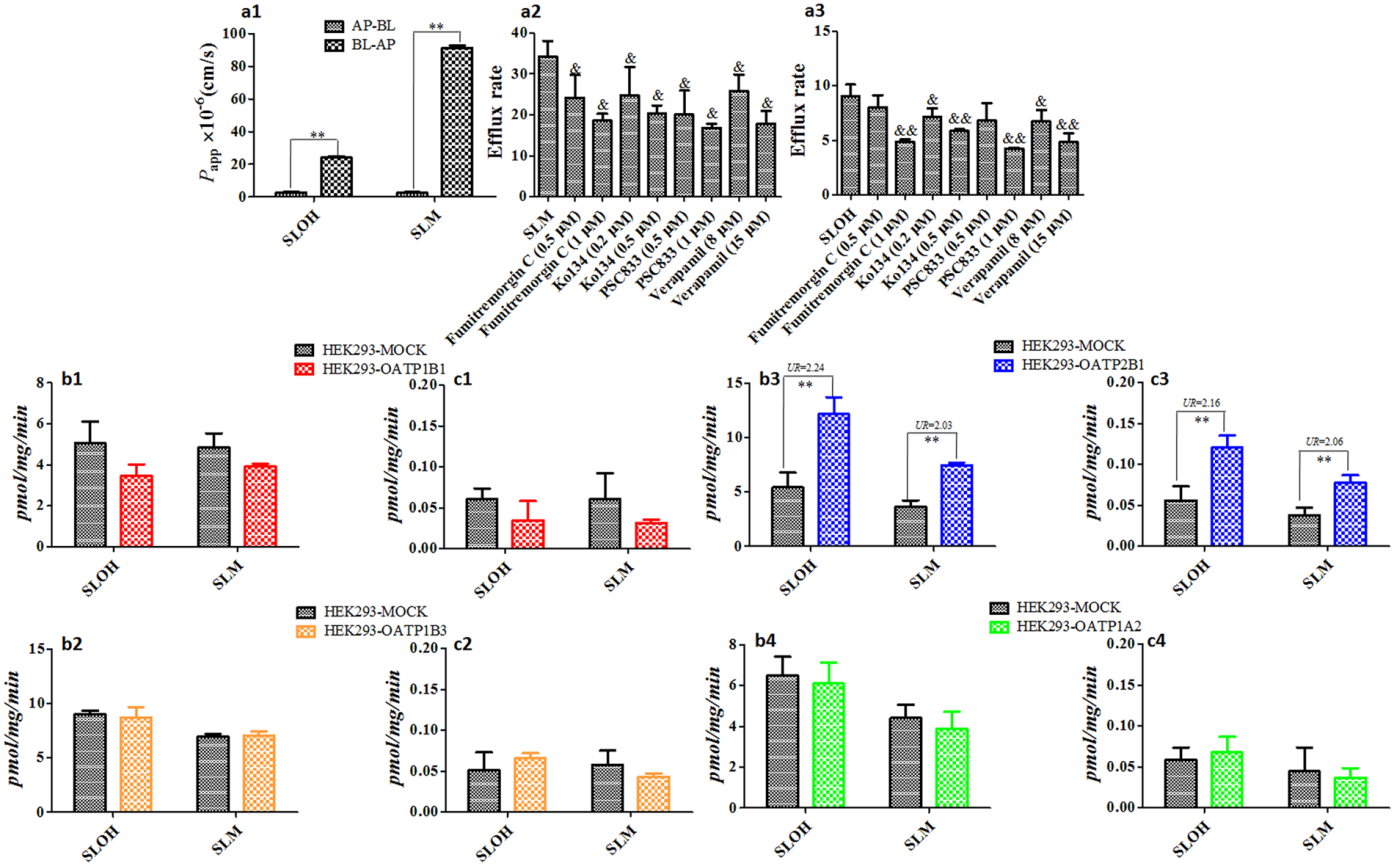
Identification of SLM and SLOH as efflux transporters (P-gp and BCRP) and influx transporters (OATP1B1/1B3/2B1/1A2) substrates. (**a1**) The transportation in apical-to-basolateral and basolateral-to-apical directions in Caco-2 cell. (**a2**,**a3**) The efflux ratio in the presence or absence of P-gp and BCRP specific inhibitors in Caco-2 cell; The transportation comparison between transfected HEK293 cells and mock groups, the final concentration of SLOH and SLM were 10 μM (**b1**–**4**) and 1 μM (**c1**–**4**). Statistical significance in parameters obtained from two groups was estimated using Student’s t-test and obtained from multiple groups was evaluated using One-way ANOVA followed by Dunnett’s test. *p < 0.05, **p < 0.01, ^&^ *p* < 0.05 and ^&&^ *p* < 0.01 (*n* = 3, Mean ± SD).

As shown in Fig. 2(a1– 3), the mean *P*_app_ values of SLOH and SLM (1 μM) in Caco-2 cells in the apical-to-basolateral (A → B) direction were 2.70 × 10^−6^ and 2.50 × 10^−6^ cm/s, respectively, indicating relatively low intestinal absorption rates. The mean efflux ratios of SLOH and SLM were found to be 9.00 and 34.0, respectively. The high efflux ratios were reduced in the presence of PSC833 and verapamil (P-gp specific inhibitors) as well as fumitremorgin C and Ko134 (BCRP specific inhibitors), and the efflux ratio reductions were found to be concentration-dependent. Moreover, we found the *V*_u,brain_ values in brain slices model of SLOH and SLM were improved in the presence of P-gp and BCRP inhibitors (Fig. S13). These observations suggested that SLOH and SLM were the substrates of P-gp and BCRP, and blocking the P-gp or BCRP functions could enhance their accumulation in the brain.

### OATP1B1/1B3/2B1/1A2 substrates identification

As shown in Fig. 2(b1– 4,c1-4), there were no significant differences in uptake for SLOH and SLM by OATP1B1, OATP1B3 and OATP1A2 transfected HEK293 versus WT cells. The uptake was found to be doubled in cells transfected with OATP2B1. The results indicated that SLOH and SLM were the substrates of OATP2B1, an important uptake SLC transporters in hepatocyte and brain endothelial cell.

### Metabolic stability studies

In mice or human liver microsomal preparations supplemented with UDPGA and alamethacin, no reduction (*t*_1/2_ > 500 mins, 0.5 mg/mL microsomes protein) was observed, indicating glucuronidation was not a primary metabolic pathway for SLOH and SLM. Liver microsomal incubations supplemented with NADPH (Table 2) showed moderate elimination in mice and high reduction in human for SLOH and SLM, but the microsomes clearance scaled (*Cl*_hep_) by the well-stirred venous equilibration liver model (with correction for both plasma and microsomes binding) were found to be low, mainly resulting from high plasma binding. Owning to the hepatocytes uptake affected by OATP2B1, which was located on the sinusoidal membrane of hepatocytes (Fig. 2(b1– 4,c1-4)), the hepatic clearance scaled (*Cl*_hep_) was found to be comparable to that scaled from microsomes data.

**Table 2:** Liver microsomes stability and hepatocyte stability in mice and human for SLOH and SLM (*n* = 3, Mean ± SD).

Parameters	SLOH		SLM	
	Mouse	Human	Mouse	Human
Microsomes *Cl*_int,app_ (mL/min/mg)	0.0300 ± 0.0000	0.0700 ± 0.0100^$$^	0.0300 ± 0.0000	0.110 ± 0.0200^$$^
Microsomes *Cl*_u, int,app_ (mL/min/mg)	0.170 ± 0.0200^$$^	1.00 ± 0.160^$^	0.300 ± 0.0200^$$^	1.50 ± 0.200^$^
Microsomes *Cl*_int,scaled_ (mL/min/Kg)	110 ± 13.0	82.0 ± 13.0^$$^	130 ± 9.00	140 ± 19.0^$$^
Microsomes *Cl*_u, int,scaled_ (mL/min/Kg)	680 ± 75.0^$$^	120 × 10 ± 200^$^	120 × 10 ± 82.0^$$^	190 × 10 ± 260^$^
Microsomes scaled *Cl*_hep_ (L/h/Kg)	1.10 ± 0.100	0.120 ± 0.0200	0.960 ± 0.0500	0.100 ± 0.0100
Hepatocyte scaled *Cl*_hep_ (L/h/Kg)	0.580 ± 0.100	0.0600 ± 0.0100	0.520 ± 0.100	0.0500 ± 0.0100

Statistical significance in parameters was estimated by Student’s t-test. ^$^ *p* < 0.05 and ^$$^ *p* < 0.01, (SLOH *vs* SLM).

Consistent with these findings, qualitative examination of metabolites in microsomes revealed fifteen phase I oxidative metabolites for SLOH (M2-M16) (Table S1 and Figs 1, SI-5), and ten phase I oxidative metabolites (M2-M11) for SLM (Table S2 and Figs 1, S1 and S6-9). The metabolites were mainly derived from dealkylation, dehydration, hydroxylation and carbonylation.

### Contribution of individual CYP to oxidation

**Figure 3:**
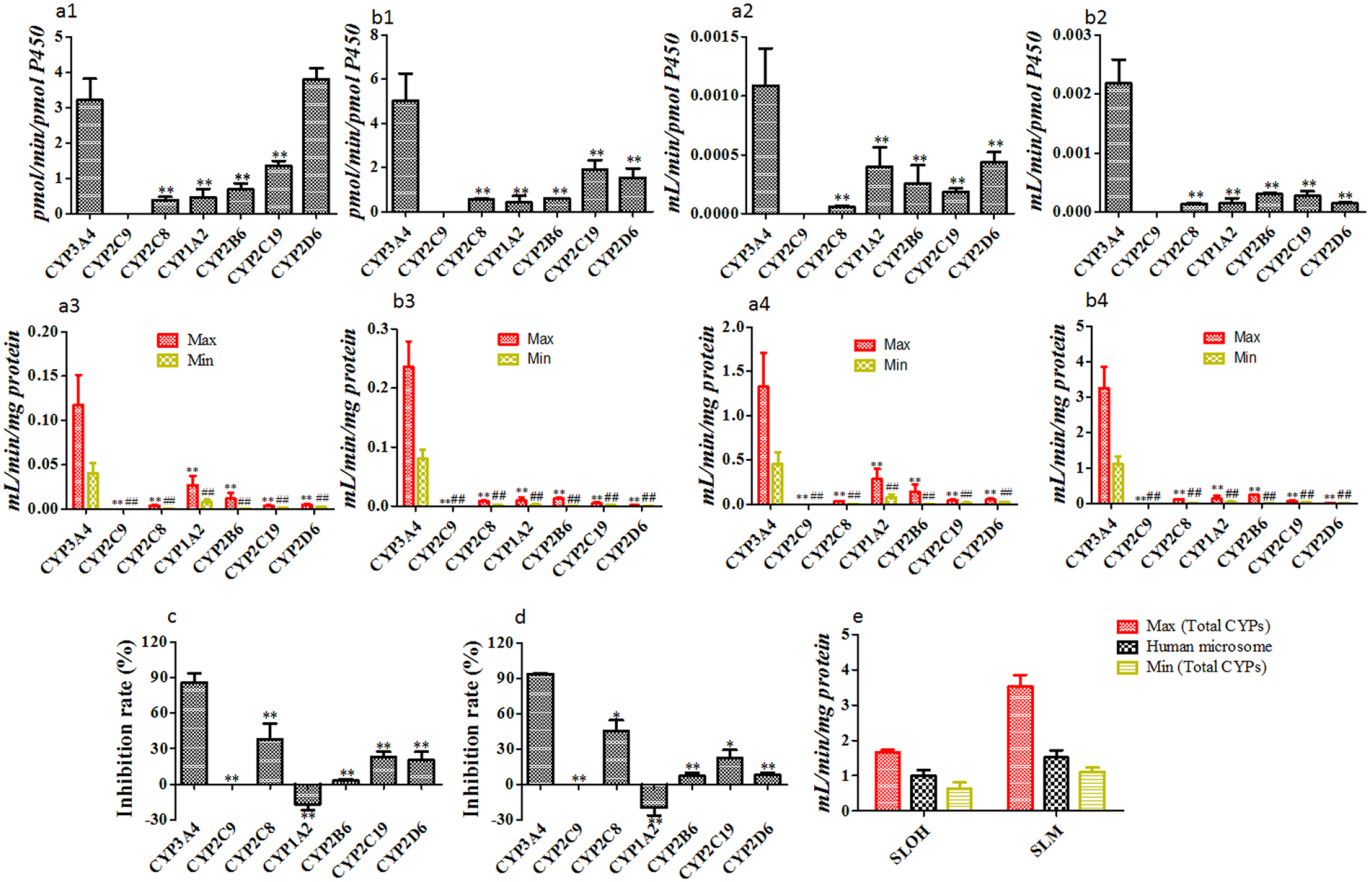
Metabolic phenotyping by recombinant CYPs and by chemical inhibition models. *V*_max_ values of SLOH (**a1**) and SLM (**b1**) metabolism in recombinant CYPs; *V*_max_/*K*_m_ values of SLOH (**a2**) and SLM (**b2**) in recombinant CYPs; **a3** (SLOH) and b3 (SLM) represent the bound recombinant CYPs clearance scaled to minimum or maximum expression values of different CYPs in human liver microsomes; a4 (SLOH) and **b4** (SLM) represent normalized unbound recombinant CYPs clearance; (**c**) (SLOH) and (**d**) (SLM) represent metabolic inhibition rate in human liver microsomes incubated with different chemical inhibitors; The total unbound recombinant CYPs clearance of SLOH (**a4**) and SLM (**b4**) were plotted with microsome metabolism in (e) panel. Statistical significance in parameters obtained from multiple groups was estimated using One-way ANOVA followed by Dunnett’s test. (*vs* CYP3A4 group) *p < 0.05, **p < 0.01, ^#^ *p* < 0.05, and ^##^ *p* < 0.01 (*n* = 3, Mean ± SD).

As shown in Fig. 3(a2 and b2), the kinetics parameter (*V*_max_/*K*_m_) value of the compounds in recombinant CYP3A4 was significantly higher than that in other recombinant CYPs. When we scaled *in vitro* clearance data based on the average expressions of specific CYPs in the human liver microsomes^13,14^, the bound or unbound metabolic clearance in CYP3A4 was accounted for up to 70% for SLOH and 90% for SLM of the total CYPs clearance (Fig. 3(a3,b3,a4 and b4)). Besides, the metabolic rates in human liver microsomes were significantly affected by the CYPs specific inhibitors, among which, specific CYP3A4 inhibitor such as ketoconazole showed the highest inhibiting rate (Fig. 3(c and d)). In addition, the total unbound recombinant CYPs clearance (pmol/mg protein) scaled to minimum expression values of different CYPs in human liver microsomes^13,14^ was closer to the unbound intrinsic clearance in human liver microsomes (Fig. 3(e)). Taken together, our data suggested that oxidative reactions of SLOH and SLM by CYP3A4 were the major metabolic pathway in human liver microsomes.

### Systemic clearance after a single dose to mice aging 2 months

As shown in Table 3, the plasma clearance was low, about 0.710 L/h per Kg for SLM and 0.610 L/h per Kg for SLOH. The unbound renal clearance (*Cl*_u, renal_) values in mice was 1.10 L/h per Kg for SLM and 0.160 L/h per Kg for SLOH, which were in line with the glomerular filtration rate (GFR) (0.600 L/h per Kg)^15^, suggesting the lack of active tubular secretion. The *Cl*_bile_ was 0.400 L/h per Kg for SLM and 0.320 L/h per Kg for SLOH, indicative of high biliary clearance compared with plasma clearance.

**Table 3:** Systemic clearance of SLOH and SLM in C57BL/6 mice aging 2 months after *i.v*. 1 mg/kg dosage (*n* = 5, Mean ± SD).

Compound	*Cl*_p_ (L/h per Kg)	Total *Cl*_bile_ (L/h per Kg)	Total *Cl*_renal_ (L/h per Kg)	*Cl*_u, renal_^a^ (L/h per Kg)	*Cl*_u, renal_^a^/GFR Ratio
SLM	0.710 ± 0.130	0.330 ± 0.0200	0.0400 ± 0.0100	1.10 ± 0.230	1.90 ± 0.390
SLOH	0.610 ± 0.140	0.320 ± 0.0200	0.0100 ± 0.0000^$$^	0.160 ± 0.0300^$$^	0.260 ± 0.0400^$$^

^a^ *Cl*_u, renal_ in blood was estimated by normalizing total *Cl*_renal_ with the unbound free fraction in blood, which in turn was obtained by dividing the unbound plasma free fraction by the blood to plasma ratio. GFR value in mice is 0.6 L/h per Kg. Statistical significance in parameters was estimated by Student’s T-test. ^$^ *p* < 0.05 and ^$$^ *p* < 0.01, (SLOH *vs* SLM).

### Systemic clearance and brain distribution in WT and 3 × Tg-AD mice (8 months)

As shown in Table 4 and Fig. S11, there were no significant differences in the systemic parameters between WT and AD mice. The plasma clearance was approximately 0.600 L/h per kg for SLOH and 0.700 L/h per kg for SLM, which were similar to that obtained in C57BL/6 mice of 2 months of age. The unbound interstitial fluid to plasma ratio (*K*_puu,brain_) was about 8.10 for SLOH and 11.0 for SLM, which favored brain entry. Remarkably, the *K*_puu,brain_ value in AD mice was 8–fold higher for SLOH and 4-fold higher for SLM than that in WT mice, which was probably caused by the low expression of P-gp, an efflux transport highly expressed at the luminal side of the brain endothelial cells in AD mice^16^.

**Table 4:** Classic and CNS pharmacokinetics parameters of SLOH and SLM in WT and AD mice (8 months) after 1 mg/kg *i.v*. administration (*n* = 3, Mean ± SD).

Parameters	SLOH		SLM	
	**WT**	**AD**	**WT**	**AD**
*K*_in_ (μL/min per g brain)	1.10 ± 0.100	4.00 ± 0.500**	1.70 ± 0.630	4.10 ± 1.10*
*K*_puu,brain_	0.980 ± 0.220	8.10 ± 0.290**	2.50 ± 0.440	11.0 ± 2.40**
*Cl*_p_ (L/h per Kg)	0.450 ± 0.190	0.600 ± 0.190	0.680 ± 0.0300	0.790 ± 0.150
Vd_ss_ (L/Kg)	2.10 ± 0.450	2.30 ± 0.710	1.30 ± 0.580	1.30 ± 0.320
*t*_1/2_ (h)	4.30 ± 1.10	4.80 ± 1.20	6.00 ± 1.00	3.50 ± 1.00
*MRT*_0−∞_	3.00 ± 1.20	2.90 ± 0.380	1.50 ± 0.180	1.70 ± 0.550

Statistical significance in parameters was estimated by Student’s t-test. *p < 0.05 and **p < 0.01, (WT *vs* AD).

## Discussion

Overall distribution of the compounds after *i.v*. have been examined. The low to moderate *Vd*_ss_ values (1–2.5) indicated that the compounds had little chance to accumulate in peripheral tissues after multiple-dosing (Tables S3 and 4). SLOH and SLM in *i.v*. dosing to mice ranged from 0.2 mg/kg to 4 mg/kg, which showed linearity kinetics dependent on dose. The relatively low plasma clearance was likely due to high plasma binding (Tables S3, 4 and Fig. S10). The plasma clearance for SLOH (≈0.900 L/h per kg) composed of 34.0% biliary clearance, 1.10% renal clearance and the remaining 64.0% clearance, which matches well with hepatic clearance scaled from hepatocyte measurement (Tables 2 and 3). The plasma clearance for SLM (≈0.890 L/h per kg) composed of 38.0% biliary clearance, 4.50% renal clearance and presumably 58.0% hepatic clearance, which is also in good accordance with the hepatic clearance scaled from *in vitro* data (Tables 2 and 3). Liver first pass elimination then accounted for 16.0% and 16.0% liver blood flow for SLOH and SLM, respectively. The biliary clearance was relatively high, which was due to the bile efflux by P-gp and BCRP (the major active canclicular transporters). Besides, metabolic stability studies (Fig. 1 and Table 2) showed that oxidation in microsomes were the major hepatic clearance pathway. Metabolic phenotyping studies using both recombinant CYPs and chemical inhibition (Fig. 3(a1,b1,a2,b2,c and d)) showed that CYP3A4 was the most important enzyme in the oxidation of SLOH and SLM in CYPs. The total unbound recombinant CYPs clearance (pmol/mg protein) scaled to minimum expression values of different CYPs in human liver microsomes^13,14^ was found to be closer to the unbound intrinsic clearance in human liver microsomes (Fig. 3(e)). Taken together, oxidation by CYP3A4 was the major hepatic clearance pathway in human. It has been reported that the mouse CYP3A11 is the most similar to human CYP3A4 (76% amino acid homology)^17,18^. Hence hepatic clearance in mice might be mainly attributed to the metabolism of SLOH and SLM by CYP3A11.

For SLOH or SLM to elicit the desirable pharmacodynamics effects, it should be able to pass brain endothelial cells. It is well documented that the luminal membrane of brain endothelial cells highly express efflux transporters, such as P-gp and BCRP, *etc*., and influx transporters (OATP2B1/1A2)^19,20^. We have demonstrated that SLOH and SLM were affected by the efflux transporters (P-gp and BCRP) in Caco-2 cell study and uptake transporter (OATP2B1) in stably transfected HEK293 cells study (Fig. 2). We also found that OATP2B1 contributed to A→B transport through Caco-2 cell (Fig. S12), although a *P*_app_ value of approximately 2.00 × 10^−6^ cm/s represented low overall permeation in the intestinal model. Brain slice study of SLOH and SLM displayed their preferred intracellular distribution once entered brain, and the distribution were improved in the presence of P-gp and BCRP inhibitors, which was consistent with the previous report that glia cells (microglia and astrocytes) highly expressed efflux transporters (P-gp and BCRP), lowly expressed OATP2B1 (Fig. S13)^21^. However, highly expressed OATP2B1 on brain endothelium was responsible for the uptake of the compounds from systemic circulation. The *K*_puu_,_brain_ values in AD mice were 8.10 for SLOH and 11.0 for SLM, significantly over 1, which indicated that compounds penetration brain endothelium was affected predominantly by OATP2B1 rather than by P-gp and BCRP. It is noteworthy that the values in AD mice were 5–8 folds higher than that in WT mice, probably due to the down-regulated expression/dysfunction of P-gp at the brain endothelium in AD disease^16,22,23^. Overall, our results suggested that SLM and SLOH could reach the ISF in AD mice effectively with the aid of OATP2B1 influx. *C*_u,brain_ and unbound *AUC*_0−t_ in ISF, the most important parameters pertained to CNS drug brain bioavailability, can be calculated from *C*_plasma_, *f*_u,plasma_, *AUC*_plasma_ and *K*_puu_ parameters. We also predicted the oral bioavailability of the compounds to be around 10%, by combination of intestinal absorption (derived from Caco-2 cell permeability^24^) and the liver first pass elimination.

## Conclusions

**Figure 4:**
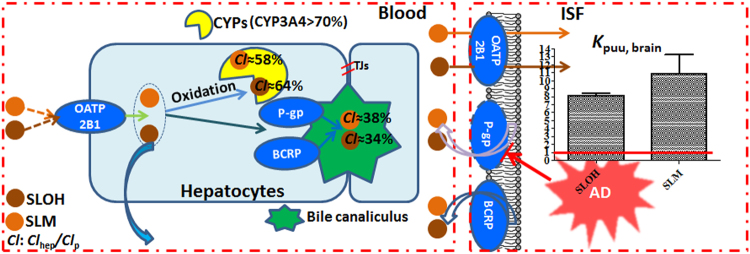
Schematic summary of systemic clearance and brain distribution of SLOH and SLM.

This study provides key information regarding the systemic clearance and brain disposition characteristics of SLOH and SLM, the novel beta-amyloid peptide aggregation inhibitors for AD treatment (Fig. 4). The plasma clearance of the compounds was relatively low, which was mainly composed of hepatic clearance and biliary clearance. The efflux by P-gp and BCRP located in the canalicular membrane of hepatocytes resulted in high biliary clearance. Ten phase I oxidative metabolites for SLM and fifteen phase I oxidative metabolites for SLOH in microsomes incubation were identified in which the oxidative reactions by CYP3A4 were the major hepatic clearance pathways. The permeability of compounds through brain endothelium was affected by both efflux transports (P-gp and BCRP) and influx transport (OATP2B1), of which influx was the major effector, as judged by large *K*_puu_ values. Moreover, it has been shown that the transportation of these compounds was greatly facilitated in AD mice than that in WT animals. The work set a framework for further PK/PD studies on this family of compounds.

## Materials and Methods

### Ethics Statement

All procedures involving animals were approved by the University of Macau Animal Ethics Committee (protocol nos.: UMAEC-15-2015, UMAEC-26-2015).

All procedures were performed in accordance with the relevant guidelines and regulations set out by the University of Macau Animal Ethics Committee.

### Chemicals and Materials

Commercially available chemicals were used in the analytical studies. SLOH and SLM were synthesized as previously described^8^. Dulbecco’s modified Eagle’s medium (DMEM), fetal bovine serum (FBS), sodium pyruvate, MEM Non-Essential Amino Acids, 0.25% trypsin-EDTA, penicillin-streptomycin and L-glutamine were obtained from Gibco BRL Co. (Gaithersburg, MD, USA). Pierce^TM^ BCA protein assay kit was purchased from ThermoFisher (Waltham, MA). *InVitro* GRO^TM^ HT Medium was purchased from BioreclamationIVT. (Baltimore, MD, USA). Transwell for 6 well plates (3450) was purchased from Corning Inc. Rapid equilibrium dialysis (RED) device was purchased from ThermoFisher (Waltham, MA). The human colorectal cancer cell lines (Caco-2, HCT116) were bought from ATCC (HTB-37TM). Pooled male ICR/CD-1 mouse microsomes and cryopreserved male ICR/CD-1 mouse hepatocytes (pool of 10 donors) were purchased from Celsis (Chicago, IL). Pooled male Mongolia human liver microsomes and cryopreserved male Mongolia human hepatocytes (pool of 11 donors) were purchased from Research Institute for Liver Diseases Co. Limited (Shanghai, China). Recombinant CYP1A2, CYP2B6, CYP2C8, CYP2C9, CYP2C19, CYP2D6, CYP3A4/5 and bactosome control were purchased from Cypex Limited (Scotland, UK). Pooled human and mouse blood (pool of 10 donors) were purchased from Shanghai Yuanmu Biotechnology CO., Limited (Shanghai, China). Pooled human and mouse plasma were purchased from BioreclamationIVT. (Baltimore, MD, USA).

### Blood cell partitioning

The blood to plasma ratio was determined using the method shown in supplemental information (Supplemental Methods). Chlorthalidone was used as a positive control in this assay (Table S4).

### Plasma, liver microsomes and hepatocyte binding by RED device

Nonspecific binding in plasma (*f*_u, plasma_), microsomes (*f*_u, mic_) and hepatocyte (*f*_u, hep_) were determined using the method seen in supplemental information (Supplemental Methods).

### Brain distribution by brain slices experiment

The unbound volume of distribution in the brain (*V*_u, brain_) was determined in fresh brain slices using the protocol published by Friden *et al*.^25^. Verapamil and gabapentin were used as positive controls in the studies (Table S4).

### *In vitro* permeability study using Caco-2 cell

The protocols for Caco-2 cells culturing and transport were described previously^26^. PSC833 (0.5 and 1 μM) and verapamil (8 and 15 μM) as P-gp specific inhibitors^27,28^, fumitremorgin C (0.5 and 1 μM) and Ko134 (0.2 and 0.5 μM) as BCRP specific inhibitors^29^ were employed to assess whether SLOH and SLM are apical efflux transporters (P-gp and BCRP) substrates. Several known marker compounds were assayed as quality control (Table S5).

The following equations (1 and 2) were used to calculate the apparent permeability coefficient (*P*_app_) value and the efflux ratio between *P*_app_ values in both transport studies (A → B and B → A).

1 $${P}_{app}=\frac{dQ}{dt}\times {{c}_{o}}^{-1}\times are{a}^{-1}$$

2 $$Efflux\,ratio=\frac{{P}_{app}(B\to A)}{{P}_{app}(A\to B)}$$

where d*Q*/d*t* (μg/S) is the linear appearance rate; *C*_0_ (μg/mL) is measured initial concentration in donor compartment; and *area* is the membrane surface area of cell monolayer (area = 4.67 cm^2^ for 6-well plate).

### OATP1B1/1B3/2B1/1A2 substrates identification studies

Human embryonic kidney (HEK) 293 cells (passage: 5–15) were stably transfected with the OATP1B1, 1B3 and 2B1 (important superfamily of solute carrier (SLC) transporters in hepatic drug disposition)^30^ and OATP1A2 and 2B1 (important uptake SLC transporters in brain endothelium)^19^. Cells were cultured in high glucose DMEM with 10% FBS, 1% penicillin-streptomycin, and seeded onto 24-well poly-D-lysine-coated plates at a density of 2.5 × 10^5^ cells/well and incubated at 37 °C, 5% CO_2_, and 95% humidity. Daily changes of medium were performed and on the second day after seeding using medium containing 10 mM sodium butyrate. At 72 h after plating, uptake experiments were conducted.

Stably transfected HEK cells and control vector-transfected HEK293 cells were first washed (3 × 1 mL) with prewarmed HBSS (pH 7.4) and then were incubated with 250 μL of HBSS containing compounds at concentrations of 1 and 10 μM. Cells were incubated for 10 mins at 37 °C for all experiments. After incubation, uptake was stopped by aspirating the incubation solution and washing each well 3 times with 200 μL of ice-old HBSS. The cells were lysed in 150 μL of 1 M NaOH for 30 mins, and then neutralized by adding equal volumes of 1 M HCl. Some of cell lysates were used as protein concentration determination by BCA method and the other were used for quantification by LC-MS/MS (Analytical method seen in support information). β-Estradiol 17-β-D-glucuronide (uptake ratio of OATP1B1: 45 ± 7.6, uptake ratio of OATP1B3: 10 ± 3.0) and estrone-3-sulfate (uptake ratio of OATP2B1: 13 ± 2.9, uptake ratio of OATP1A2: 18 ± 3.7 in this assay) were used as positive control.

The following equations (3 and 4) were used to calculate the uptake rates (*U*, pmol/mg/min) and the OATP1B1, OATP1B3, OATP2B1 and OATP1A2 uptake ratio (*UR*):

3 $$U=\frac{{C}_{lysate}}{P\times T}$$

4 $$UR=\frac{{U}_{{\rm{OATP}}1{\rm{B}}1/1{\rm{B}}3/2{\rm{B}}1/1{\rm{A}}2}}{{U}_{{\rm{HEK}}293-{\rm{MOCK}}}}$$

where *C*_lysate_ is the compound concentration in cell lysates. *P* is the protein concentration in cell lysates. *T* is the incubation time. *U*_OATP1B1/1B3/2B1/1A2_ is the uptake rate obtained in HEK293 stably transfected OATP1B1, OATP1B3, OATP2B1 or OATP1A2 transportation. *U*_HEK293-MOCK_ is the uptake rate obtained in control vector-transfected HEK293 transportation.

### *In vitro* stability in liver microsomes and hepatocytes

Microsomes and hepatocytes stability procedures and their parameters (the apparent intrinsic clearance (*Cl*_int,app_), unbound apparent intrinsic clearance (*Cl*_u, int,app_), scaled intrinsic clearance (*Cl*_int,scaled_), unbound scaled intrinsic clearance (*Cl*_u, int,scaled_), and *Cl*_hep_) calculation methods were shown in supplemental information (Supplemental Methods). The metabolites identification methods including LC-MS conditions was also found in supplemental information (Supplemental Methods). Several marker compounds as quality control were assayed (Table S6).

### Contribution of individual CYP to oxidation

Metabolism was evaluated in bactosomes prepared from bacteria expressing various human CYP isoforms. Kinetics of SLOH and SLM in bactosome containing recombinant CYP1A2, CYP2B6, CYP2C8, CYP2C9, CYP2C19, CYP2D6 and CYP3A4/5 were determined from decreasing rates at concentrations ranging from 0.25 to 32 μM. All incubations contained 50 pmol CYP. Incubation was started by adding NADPH to a final concentration of 1 mM after 5 mins of pre-incubation. Reaction was incubated for 10 mins and stopped by adding 4 folds acetonitrile (ACN) containing internal standard (IS), and the samples were analyzed by LC-MS/MS after centrifuging at 9,659 g for 5 mins (Analytical method seen in support information). The blank bactosomes from bacteria were used in parallel as controls. The profile for the compound disappearance in the presence of the expressed enzyme was corrected for any disappearance observed in the presence of the control preparation. The kinetic parameters, maximal velocity (*V*_max_) and *V*_max_/Michaelis constant (*K*_m_), were derived by fitting the experiment data with the one-component Michaelis-Menten model via regression analysis using GraphPad Prism 5.

Meanwhile, CYP isoform-specific competitive inhibitors (α-naphthoflavone (CYP1A2); orphenadrine hydrochloride (CYP2B6); montelukast sodium (CYP2C8); sulfaphenazole (CYP2C9); modafinil (CYP2C19); quinidine (CYP2D6); ketoconazole (CYP3A4)) were co-incubated at 10-fold (inhibitory constant) *K*_i_ concentration according to the FDA guideline to identify which CYPs were more likely to affect the metabolism of compounds in human microsomes^31^. Incubation conditions were carried out as mentioned in supplemental information (Supplemental Methods).

### Animal experiment

Male C57BL/6 and Bile duct-cannulated (BDC) mice (2 months, weighting: 25–30 g) obtained from Charles River, Inc. (Raleigh, NC) were used to study systemic clearance mechanism, in line with *in vitro* commercial samples from adolescent animals. Mice were fasted overnight and throughout the duration of the study (1 or 2 hours), whereas access to water was provided ad libitum. SLOH and SLM were administered *i.v*. in PBS containing 5% DMSO via the tail vein (*n* = 5) at different dosages in a dosing volume of 5 mL/kg. Serial blood samples were collected before dosing and at 0.083, 0.167, 0.25, 0.5, 1, 2, 4, 8, 12 and 24 hours after dosing. Urine samples (0–12 and 12–24 hours; pooled 0–24 hours) were also collected after *i.v*. administration (1 mg/kg). Bile samples were collected at intervals of 0–4, 4–8 and 8–24 hours (pooled 0–24 hours) after dosing (1 mg/kg) to BDC mice.

The transgenic (Tg) AD mice (B6; 129-*Psen1* ^*tm1Mpm*^ Tg (APPSwe, tauP301L) 1Lfa/Mmjax) and the corresponding wild-type (WT) (B6129SF2/J) mice was obtained from the Jackson Laboratory (Bar Harbor, Maine, USA), and maintained by the Animal Facility of Faculty of Health Sciences in University of Macau. Either 27 WT or AD mice (8 months) were randomly separated into 9 groups with 3 mice at each time point. Blood and brain were collected at time points 0.083, 0.167, 0.25, 0.5, 1, 2, 4, 8 and 16 hours after *i.v*. dosing (1 mg/kg). Mice were euthanized by isoflurane followed by cervical dislocation. All the plasma, urine, bile and brain samples were stored at −70 °C for subsequent analysis. The analytical methods for systemic and brain PK parameters were shown in supplemental information (Supplemental Methods).

### Animal parameters calculation

Parameters in animals were determined using non-compartmental analysis. The area under the plasma concentration-time curves (*AUC*) from *t* = 0 to 24 hours (*AUC*_0–24_) and t = 0 to infinity (*AUC*_0−∞_) were estimated using the linear trapezoidal rule. Systemic *Cl*_p_ was calculated as the *i.v*. dose divided by AUC_0−∞_^iv^. The terminal rate constant (*K*_el_) was calculated by a linear regression of the log-linear concentration-time curve, and the terminal elimination *t*_1/2_ was calculated as 0.693 divided by *K*_el_. Mean residence time from t = 0 to infinity (*MRT*_0−∞_) was calculated as *AUMC*_0−∞_ divided by *AUC*_0−∞_. Apparent steady-state distribution volume (*Vd*_ss_) was determined as *Cl*_p_ multiplied by *MRT*_0−∞_. The renal clearance (*Cl*_renal_) was derived as the ratio of amount (in milligrams) of SLM or SLOH in urine over the 24-hour interval postdose divided by the area under the plasma concentration-time curve (*AUC*_0-24h_). The *Cl*_bile_ was derived as the amount of (in milligrams) of SLM or SLOH in the bile over the 24-hour interval postdose/AUC_0-24h_. The influx clearance into brain (*K*_in_) was calculated by using the amount of drug in the brain (homogenate) at time *t* (*A*, brain (*t*)) (corrected for the amount of drug remaining in the cerebral vasculature) divided by plasma exposure up to this time point (*AUC*, plasma (*0* − *t*)). The *C*_u,brain_ was calculated by *A*_brain_ (homogenate, ng/g brain) divided by *V*_u,brain_ (mL/g brain). The unbound ISF to plasma ratio (*K*_puu,brain_) was calculated by unbound *AUC*_0−t_ of ISF (ng/mL × min) divided by unbound *AUC*_0–t_ (ng/mL × min) of plasma.

### Statistical analysis

Statistical significance in parameters obtained from two groups was estimated using Student’s t-test and obtained from multiple groups was evaluated using one-way ANOVA followed by Dunnett’s test. A *p* value of less than 0.05 was considered to be significantly different. All data was expressed as mean ± SD.

## Electronic supplementary material

Supporting information
