# Supplementary material for: Systemic clearance and brain distribution of carbazole-based cyanine compounds as Alzheimer’s disease drug candidates
Source: Sci Rep. 2017 Nov 27;7:16368. doi: 10.1038/s41598-017-16635-4 (PMC5703966; doi:10.1038/s41598-017-16635-4)
Supplement: Supplementary file 1 — Supporting information [file 41598_2017_16635_MOESM1_ESM.pdf]

Supplemental Data

Systemic clearance and brain distribution of carbazole-based cyanine compounds as Alzheimer's disease drug candidates

Wei Zhou, Xiaohui Hu, and Kin Yip Tam*

Faculty of Health Sciences, University of Macau, Macau SAR, PR China.

Supplemental Figures and Tables legends

Table S1 Mass spectral characteristics of the *in vitro* metabolites of SLOH.

Table S2 Mass spectral characteristics of the *in vitro* metabolites of SLM.

Table S3 Systemic pharmacokinetics parameters of SLOH and SLM in C57BL/6 mice aging 2 months after *i.v.* administration (n=5, Mean ± SD). Statistical significance in parameters was estimated by Student's t-test. (SLOH *vs* SLM group), $^{\$}p < 0.05$ and $^{\$\$}p < 0.01$ (n=5, Mean ± SD).

Table S4 Blood cell partitioning, non-specific plasma protein binding, microsomes binding, and brain distribution using brain slices of positive controls (n=3, Mean ± SD).

Table S5 The P_{app} and efflux ratio values of positive controls in Caco-2 cell transport (n=3, Mean ± SD).

Table S6 Liver microsomes stability and hepatocyte stability in mice and human of positive controls (n=3, Mean ± SD).

Fig. S1 Representative MS/MS spectrum of SLOH and SLM.

Fig. S2 Representative SIM chromatograms for T_{30min} incubations of SLOH in liver microsomes supplemented with NADPH.

Fig. S3 Representative MS spectrum for T_{30min} incubations of SLOH in liver microsomes supplemented with NADPH.

Fig. S4 Representative MS/MS spectrum for T_{30min} incubations of SLOH in liver microsomes supplemented with NADPH.

Fig. S5 The fragment ions characteristics of SLOH metabolites for T_{30min} incubations in liver microsomes supplemented with NADPH.

Fig. S6 Representative SIM chromatograms for T_{30min} incubations of SLM in liver microsomes supplemented with NADPH.

Fig. S7 Representative MS spectrum for T_{30min} incubations of SLM in liver microsomes supplemented with NADPH.

Fig. S8 Representative MS/MS spectrum for T_{30min} incubations of SLM in liver microsomes supplemented with NADPH.

Fig. S9 The fragment ions characteristics of SLM metabolites for T_{30min} incubations in liver microsomes supplemented with NADPH.

Fig. S10 The pharmacokinetics profiles in C57BL/6 mice aging 2 months. A: SLOH, B: SLM. A1 and B1 represent pharmacokinetics profiles; A2 and B2 represent linearity analysis between AUC and dosages (n=5, Mean ± SD).

Fig. S11 The pharmacokinetics and brain exposure in WT and transgenic AD model mice. A: SLOH, B: SLM. A1 and B1 represent pharmacokinetics profiles; A2 and B2 represent brain exposure in ISF. Statistical significance in parameters was estimated by Student's t-test. (*vs* WT group), $^{*}p < 0.05$ and $^{**}p < 0.01$ (n=3, Mean ± SD).

Fig. S12 The efflux ratio values of SLOH and SLM in the presence or absence of specific OATP2B1 inhibitor (Erlotinib) in Caco-2 cell transport. Statistical significance in parameters obtained from multiple groups was estimated using One-way ANOVA followed by Dunnett's test. (*vs* control group), $^{\&}p < 0.05$ and $^{\&\&}p < 0.01$ (n=3, Mean ± SD).

Fig. S13 The $V_{u,brain}$ values of SLOH and SLM in the presence or absence of P-gp and BCRP inhibitor in brain slice experiment. Statistical significance in parameters obtained from multiple groups was estimated using One-way ANOVA followed by Dunnett's test. (*vs* control group), $^{*}p < 0.05$ and $^{**}p < 0.01$ (n=3, Mean ± SD).

Supplemental Methods

Blood-to-plasma ratio

Aliquots of fresh whole blood and reference plasma (C_{CP}) are spiked with SLOH and SLM (1 μM) and incubated for an hour at 37 ^{O}C. At the end of the incubation period, plasma (C_P) is separated from whole blood by centrifugation and all plasma samples are analyzed after protein precipitation using 2 folds acetonitrile (ACN) containing berberine chloride as internal standard (IS) by LC-MS/MS (Analytical method seen below). The blood cell partitioning was calculated as follows.

$$K_{WB/P} = \frac{C_{CP}}{C_P} \quad (1)$$

Plasma, liver microsomes and hepatocyte binding by RED device

The stability of the compounds in plasma, liver microsomes and hepatocyte were studied to ensure the reliability of the binding data. Hepatocyte was deactivated by leaving them on a bench for 2 days and going through a freeze thaw cycle thereafter. This rendered the enzymes inactive but not totally denatured. Testosterone was used as a control for CYP3A to ensure no turnover, so that the enzymes were inactivated. All validation compounds were incubated at 37 ^{o}C over 4 h, and in triplicate. For plasma stability, 10 mM DMSO solutions of SLM and SLOH were diluted to 40 μM (10% DMSO). Plasma were thawed and 594 μL aliquots dispensed into a 1.5 mL tube, and then pre-warmed at 37 ^{o}C in a shaking water bath (60 oscillations/min approx.). The assay was started by addition of 6 μL aliquots to the plasma (final DMSO incubation concentration 0.1%). For liver microsomes without cofactors and deactivated hepatocyte stability, all validation compounds (1 μM) in deactivated liver microsomes (0.5 mg/mL protein concentration, same as the assay condition) or deactivated hepatocytes (0.5 million cells/mL, same as the assay condition) were added to the donor wells. 10 mM DMSO solutions of SLM and SLOH were diluted to 50 μM (10% DMSO). 490 μL aliquots microsomes (0.51 mg/mL) and hepatocytes (0.51 million cells/mL) were dispensed into a 1.5 mL tube, and then pre-warmed at 37 ^{o}C in a shaking water bath. The assay was started by addition of 10 μL aliquots of compound solutions to the corresponding biological matrices (final DMSO incubation concentration 0.2%, microsomes concentration 0.50 mg/mL, hepatocytes concentration 0.5 million cells/mL). At 0, 15, 30, 60, 120, 180 and 240 min, 50 μL plasma, microsomes or hepatocytes after protein precipitation were analyzed by LC-

MS/MS. Natural logarithm of peak area ratios (compound peak area normalized by IS peak area) were plotted against time and half-life calculated.

The spiked plasma solutions, microsomes and hepatocytes (400 μL) after observing stability were placed into the sample chamber (indicated by the red ring) with 400 μL of PBS into the adjacent chamber. The plate was sealed with a self-adhesive lid and incubated at 37 °C on an orbital shaker (1.5 g approx.) for 4 h. Aliquots (100 μL) were removed from each side of the insert, and analyzed after protein precipitation using 4 folds acetonitrile (ACN) containing berberine chloride as internal standard (IS) by LC-MS/MS (Analytical method seen below). The experimental procedure outlined in the Thermo Scientific RED device systems brochure was adopted in the present study. The several positive controls were assayed (Table S4).

***In vitro* stability in liver microsomes and hepatocytes**

Half-life ($t_{1/2}$) of SLM or SLOH in liver microsomes was determined in duplicate. In phase I reaction, the compound (1 μM) was incubated with liver microsomes (0.5 mg/mL) in 100 mM sodium phosphate buffer (pH 7.4) including 81 mM Na_2HPO_4 and 19 mM NaH_2PO_4) at 37 °C, the reaction mixture was prewarmed at 37 °C for 2 minutes before adding NADPH (1.0 mM). In phase II reaction, the compound (1 μM) was incubated with liver microsomes (0.5 mg/mL) in 100 mM Tris-HCl buffer (pH 7.4) containing 5 mM $MgCl_2$, 10 μg/mL alamethicin, and 2% (*w*/*v*) BSA. After pre-incubated on ice for 15 min, the reactions were initiated with the addition of UDPGA (5.0 mM). Aliquots of the reaction mixture at 0, 5, 10, 15, 30 or 45 minutes were added to 4 folds acetonitrile (ACN) containing internal standard (IS), and the samples were centrifuged at 9,659 g for 5 mins before LC-MS/MS analysis of substrate disappearance. For control experiments, NADPH, UDPGA and/or liver microsomes were omitted from these incubations. For the purposes of metabolite identification studies, the concentration of SLOH and SLM in microsomal incubations was raised to 10 μM and the microsomal protein concentration increased to 3 mg/mL. *InVitro*GRO™ HT Medium was used for thawing suspension of cryopreserved hepatocytes. Incubations were conducted in a 96-well flat-bottom polystyrene plate in duplicate. Hepatocytes were suspended at 0.5 × 10^6 viable cells/mL of HT Medium and prewarmed at 37 °C for 10 minutes. Incubations were initiated with the addition of SLM and SLOH (final concentration=1 μM) and were conducted at 37 °C, 75% relative humidity, and 5% CO_2. The reaction was stopped at 0, 5, 15, 30, 60, 90, 120 minutes by the addition of 4 folds ACN containing berberine chloride as internal standard

(40 nM). The samples were centrifuged at approximately 1,900 g for 10 mins before LC-MS/MS analysis. The CYP3A4 and CYP1A2 activities in human hepatocytes were assayed by testosterone 6β-hydroxylation (141 ± 23.0 pmol/min/10^6 cells) and phenacetin *O*-deethylation (24.3 ± 3.25 pmol/min/10^6 cells). The other positive controls assays were shown in Table S6. The scaling of *in vitro* to *in vivo* clearance based on the well-stirred model [1] (equation 10) was carried out as follows (2-5: microsomes; 6-9: hepatocytes):

$$Cl_{\text{int, app}} = \frac{0.693}{t_{1/2\,(\text{min})}} \bullet \frac{\text{mL incubation}}{\text{mg microsomes}} \qquad (2)$$

$$Cl_{\text{u, int, app}} = \frac{Cl_{\text{int, app}}}{f_{\text{u, mic}}} \qquad (3)$$

$$Cl_{\text{int, scaled}} = \frac{0.693}{t_{1/2\,(\text{min})}} \bullet \frac{\text{mL incubation}}{\text{mg microsomes}} \bullet MPPGL \bullet LWPBW \qquad (4)$$

$$Cl_{\text{u, int, scaled}} = \frac{Cl_{\text{int, scaled}}}{f_{\text{u, mic}}} \qquad (5)$$

$$Cl_{\text{int, app}} = \frac{0.693}{t_{1/2\,(\text{min})}} \bullet \frac{\text{mL incubation}}{\text{million cells}} \qquad (6)$$

$$Cl_{\text{u, int, app}} = \frac{Cl_{\text{int, app}}}{f_{\text{u, hep}}} \qquad (7)$$

$$Cl_{\text{int, scaled}} = \frac{0.693}{t_{1/2\,(\text{min})}} \bullet \frac{\text{mL incubation}}{\text{million cells}} \bullet HPGL \bullet LWPBW \qquad (8)$$

$$Cl_{\text{u, int, scaled}} = \frac{Cl_{\text{int, scaled}}}{f_{\text{u, hep}}} \qquad (9)$$

Well-stirred model

$$Cl_{\text{hep}} = \frac{Q \bullet f_{\text{u, plasma}} \bullet Cl_{\text{u, int, scaled}}}{Q + f_{\text{u, plasma}} \bullet Cl_{\text{u, int, scaled}}} \qquad (10)$$

Where *MPPGL* is microsomal protein per gram liver (human: 48.8 mg/g Liver; mice: 45 mg/g Liver), *HPGL* is hepatocellularity (million cells per gram liver) (human: 99; mice: 128) and *LWPBW* is grams liver per kg body weight (human: 25.7g/kg; mice: 87.5 g/kg) [2]. $Cl_{\text{int, app}}$ is apparent intrinsic clearance, $Cl_{\text{u, int, app}}$ is unbound apparent intrinsic clearance, $Cl_{\text{int, scaled}}$ is scaled intrinsic clearance, $Cl_{\text{u, int, scaled}}$ is unbound

scaled intrinsic clearance, Cl_{hep} is plasma hepatic clearance, Q is hepatic blood flow (human: 20.7 mL/min per kg; mice: 90 mL/min per kg) [3].

LC-HR MS (LTQ-Orbitrap XL hybrid MS) conditions for metabolites identification of SLOH and SLM

LC-MS experiments were carried out on a Thermo Electron LTQ-Orbitrap XL hybrid MS (ThermoFinnigan, Bremen, Germany) equipped with an ESI interface. An Accela HPLC system (ThermoElectron) was equipped with an autosampler, a vacuum degasser unit and a quaternary pump. The MS employing positive ionization was calibrated using calibration standards mixture allowing for mass accuracies less than 5 ppm in external calibration mode. The ionization voltage was 4.2 KV and the capillary temperature was set at 300 ^{o}C. Nitrogen was used as both the sheath gas (40 units) and auxiliary gas (10 units). The resolving power was 15000 for full-scan and 7500 for the MS^2 scans. Chromatographic separation was achieved on a Luna ODS C18 column (150 × 2.1 mm, 5 μM; Phenomenex, Torrance, CA, USA). The mobile phase was composed of A (ACN containing 0.1% formic acid), B (Water containing 0.1% formic acid) using a gradient elution of 10%-10% A, 90%-90% B at 0-5 min, 10%-30% A, 90%-70% B at 5-8 min, 30%-90% A, 70%-10% B at 8-20 min, 90%-10% A, 10%-90% B at 20-21 min and hold for 4 min. The flow rate was set at 0.3 $mL{\cdot}min^{-1}$. The auto-sampler was conditioned at 4 ^{o}C and the injection volume was 5 μL.

A Thermo Xcaliber 2.1 workstation was used for the data acquisition and processing. For the computer-based MDF approach, representative structures with predicted mass defect windows were set as filtering templates for screening homologous compounds. Thermo Scientific™ MetWorks™ and Mass Frontier software automated structural elucidation.

Bio-sample preparations and LC-MS/MS (TQD) analysis for quantitation of SLOH and SLM

The LC-MS/MS condition was reported previously [4]. Briefly, under positive mode of Waters Xevo TQD, SLM (m/z 437＞347 as quantifier ion and m/z 437＞168 as qualifier ion), SLOH (m/z 467＞333 as quantifier ion and m/z 467＞154 as qualifier ion) and berberine (as internal standard, m/z 336＞320 as quantifier ion and m/z 336＞278 as qualifier ion) were simultaneously monitored. Plasma, urine and bile samples preparation method was completed as previously described [4]. For brain sample, brain was weighted and PBS buffer (pH=7.4) was added based on 10-fold volume of each brain weight. Brain samples were cut into smaller pieces before homogenization. Then 20 μL of homogenate were vortex

mixed with nitrogen-dried IS for 1 min. 40 μL of ACN was then added to precipitate the protein. After vortexed for 1.5 mins, the samples were centrifuged at 9,659 g for 5 mins. The supernatant was transferred to an autosampler vial and an aliquot of 1 μL was injected onto the LC-MS/MS system for analysis.

References

[1] Obach, R.S. Prediction of human clearance of twenty-nine drugs from hepatic microsomal intrinsic clearance data: An examination of in vitro half-life approach and nonspecific binding to microsomes. Drug Metab. Dispos. 27, 1350-1359 (1999).

[2] Barter, Z.E. et al. Scaling factors for the extrapolation of *in vivo* metabolic drug clearance from *in vitro* data: reaching a consensus on values of human microsomal protein and hepatocellularity per gram of liver. Curr. Drug Metab. 8, 33-45 (2007).

[3] Davies, B. & Morris, T. Physiological parameters in laboratory animals and humans. Pharm. Res. 10, 1093-1095 (1993).

[4] Zhou, W., Wu, X., Li, J., Hu, X. & Tam, K. Quantification of permanent positively charged compounds in plasma using one-step dilution to reduce matrix effect in MS. Bioanalysis 8, 497-509 (2016).

Table S1

Metabolite	M^+	Formula	Metabolic pathway	Retention time (t_R)	Fragment ions
Parent	467.2329	$C_{30}H_{31}N_2O_3$	N.A.	16.8	423.2074, 377.1659, 347.1551, 333.1396, 320.1310, 319.1226, 154.0654
M1	467.2329	$C_{30}H_{31}N_2O_3$	Cis-trans isomer	15.4	423.2074, 377.1645, 347.1536, 333.1380, 320.1310, 319.1226, 154.0647
M2	453.2173	$C_{29}H_{29}N_2O_3$	*O*-demethylation	16.0	409.1898, 377.1645, 347.1530, 333.1378, 320.1290, 319.1226, 154.0645
M3	483.2278	$C_{30}H_{31}N_2O_4$	Hydroxylation	15.8	439.2010, 393.1593, 363.1488, 349.1331, 336.1250, 335.1172, 154.0648
M4	483.2278	$C_{30}H_{31}N_2O_4$	Hydroxylation	16.1	365.1641, 347.1530, 333.1378, 321.1379, 319.1226, 154.0647
M5	469.2122	$C_{29}H_{29}N_2O_4$	*O*-demethylation+ hydroxylation	15.4	425.1865, 393.1604, 363.1495, 349.1341, 336.1264, 335.1186, 154.0652
M6	469.2122	$C_{29}H_{29}N_2O_4$	*O*-demethylation+ hydroxylation	15.6	407.1760, 377.1645, 333.1391, 320.1309, 319.1226, 154.0652
M7	409.1911	$C_{27}H_{25}N_2O_2$	*O*-dealkylation	16.0	377.1648, 365.1648, 347.1530, 333.1387, 320.1309, 319.1233, 154.0650
M8	425.1860	$C_{27}H_{25}N_2O_3$	*O*-dealkylation+ hydroxylation	15.5	407.1760, 377.1658, 363.1498, 333.1396, 320.1310, 319.1233, 154.0654
M9	365.1648	$C_{25}H_{21}N_2O$	*N*-dealkylation	16.4	321.1380, 319.1233, 154.0648
M10	381.1598	$C_{25}H_{21}N_2O_2$	*N*-dealkylation+ hydroxylation	15.3	337.1334, 335.1186, 154.0650
M11	381.1598	$C_{25}H_{21}N_2O_2$	*N*-dealkylation+ hydroxylation	15.6	337.1335, 335.1186, 154.0650
M12	381.1598	$C_{25}H_{21}N_2O_2$	*N-dealkylation+ hydroxylation*	16.2	363.1487, 334.1462, 320.1290, 319.1230, 167.0728
M13	407.1754	$C_{27}H_{23}N_2O_2$	*N-dealkylation+ hydroxylation+dehydration*	15.8	N.A.
M14	423.1703	$C_{27}H_{23}N_2O_3$	*O-dealkylation*+carbonylation	16.0	379.1445, 333.1389, 320.1290, 319.1235, 154.0651
M15	451.2016	$C_{29}H_{27}N_2O_3$	*O*-demethylation+*hydroxylation+dehydration*	15.8	N.A.
M16	411.2067	$C_{27}H_{27}N_2O_2$	unknown	15.8	N.A.

All metabolites were observed in liver microsomes from preclinical mice and humans. (N.A.: unidentified).

Table S2

Metabolite	M^+	Formula	Metabolic pathway	Retention time (t_R)	Fragment ions
Parent	437.2224	$C_{29}H_{29}N_2O_2$	N.A.	17.1	361.1696, 347.1541, 334.1454, 333.1376, 319.1228, 168.0807
M1	437.2224	$C_{29}H_{29}N_2O_2$	Cis-trans isomer	15.7	361.1696, 347.1541, 334.1454, 333.1376, 319.1228, 168.0807
M2	423.2067	$C_{28}H_{27}N_2O_2$	*O*-demethylation	16.3	361.1693, 347.1538, 334.1452, 333.1374, 168.0806
M3	453.2173	$C_{29}H_{29}N_2O_3$	Hydroxylation	16.3	363.1497, 335.1534, 319.1226, 168.0804
M4	439.2016	$C_{28}H_{27}N_2O_3$	*O*-demethylation+ hydroxylation	15.8	421.1917, 361.1703, 347.1548, 334.1466, 333.1388, 168.0810
M5	379.1805	$C_{26}H_{23}N_2O$	*O*-dealkylation	16.3	361.1703, 347.1545, 334.1457, 333.1379, 168.0809
M6	395.1754	$C_{26}H_{23}N_2O_2$	*O*-dealkylation+ hydroxylation	15.8	377.1646, 347.1540, 334.1460, 333.1382, 168.0807
M7	335.1543	$C_{24}H_{19}N_2$	*N*-dealkylation	16.7	319.1232, 168.0808
M8	351.1492	$C_{24}H_{19}N_2O$	*N*-dealkylation+ hydroxylation	15.9	N.A.
M9	421.1911	$C_{28}H_{25}N_2O_2$	*O*-demethylation+*hydroxylation+dehydration*	16.6	361.1703, 347.1544, 334.1460, 333.1382, 168.0811
M10	437.1860	$C_{28}H_{25}N_2O_3$	*O-dealkylation*+carbonylation	16.4	379.1810, 361.1703, 347.1545, 334.1459, 333.1381, 168.0809
M11	393.1598	$C_{26}H_{21}N_2O_2$	*O-dealkylation*+carbonylation	16.3	347.1534, 334.1451, 333.1373, 168.0804

All metabolites were observed in liver microsomes from preclinical mice and humans. (N.A.: unidentified)

Table S3

Compound	Dose (mg/kg)	$AUC_{0-\infty}$ (ng/mL*h)	Cl_p (L/h per Kg)	Vd_{ss} (L/Kg)	$t_{1/2}$ (h)	$MRT_{0-\infty}$ (h)
**SLM**	0.2	240 ± 74.0	0.890 ± 0.240	1.50 ± 0.630	6.40 ± 4.00	3.50 ± 0.950
	0.6	580 ± 170	0.930 ± 0.140	2.10 ± 0.540$	6.00 ± 1.10	3.70 ± 0.860
	1.0	140 × 10 ± 240	0.710 ± 0.130	2.20 ± 0.610$$	9.30 ± 3.30	3.20 ± 1.00
	2.0	320 × 10 ± 100× 10	0.750 ± 0.180	1.70 ± 0.550$	7.10 ± 0.550	2.50 ± 0.180$$
	4.0	500 × 10 ± 880	0.830 ± 0.160	2.10 ± 0.200	6.50 ± 2.00	3.00 ± 0.520$$
**SLOH**	0.2	220 ± 61.0	0.930 ± 0.240	1.00 ± 0.330	6.90 ± 3.70	2.70 ± 1.50
	0.6	860 ± 310	0.770 ± 0.0800	1.20 ± 0.580$	5.50 ± 1.10	2.30 ± 0.780
	1.0	230 × 10 ± 130 × 10	0.610 ± 0.140	0.830 ± 0.430$$	5.80 ± 0.160	1.80 ± 0.100
	2.0	320 × 10 ± 86.0	0.640 ± 0.0200	1.00 ± 0.360$	4.60 ± 0.760	1.80 ± 0.170$$
	4.0	620 × 10 ± 580	0.710 ± 0.130	1.00 ± 0.350	5.80 ± 1.70	1.70 ± 0.0800$$

Table S4

Parameters	Compounds	Species	
		Mouse	Human
Blood to plasma ratio	Chlorthalidone	6.20 ± 0.200	11.0 ± 3.20
Unbound plasma ($f_{u,\ plasma}$ %)	Atenolol	98.0 ± 0.210	96.0 ± 1.20
	Propranolol	0.220 ± 0.0200	6.50 ± 0.480
Unbound Liver microsomes ($f_{u,\ mic}$ %)	Atenolol	100	100
	Propranolol	76.0 ± 0.700	61.0 ± 1.60
Unbound brain distribution ($V_{u,\ brain}$ mL/g brain)	Verapamil	36.0 ± 9.70	—
	Gabapentin	2.20 ± 1.00	—

Table S5

Compounds / Parameters	P_{app} (A-B) (cm/s) × 10^{-6}	Efflux ratio
Sodium fluorescein (1 μM input)	1.40 ± 0.260	—
Rhodamine 123 (1 μM input)	0.730 ± 0.0200	—
Atenolol (1 μM input)	0.480 ± 0.0900	—
Propranolol (1 μM input)	17.0 ± 1.30	—
Verapamil (2 μM input)	23.0 ± 1.20	—
Digoxin (2 μM input)	3.50 ± 0.340	5.00 ± 1.50
Topotecan (2 μM input)	1.20 ± 0.0900	4.60 ± 1.70

Table S6

Compounds / Species	$Cl_{int, app}$ Mouse	$Cl_{int, app}$ Human
Propranolol (1 μM input) (Microsomes) (mL/min/mg)	0.0740 ± 0.00200	0.0220 ± 0.00100
Verapamil (1 μM input) (Microsomes) (mL/min/mg)	0.290 ± 0.00500	0.0690 ± 0.00300
Phenacetin (1 μM input) (Hepatocytes) (mL/min/10^6 cells)	0.0330 ± 0.00800	—
Verapamil (1 μM input) (Hepatocytes) (mL/min/10^6 cells)	0.0310 ± 0.00100	—

Fig. S1

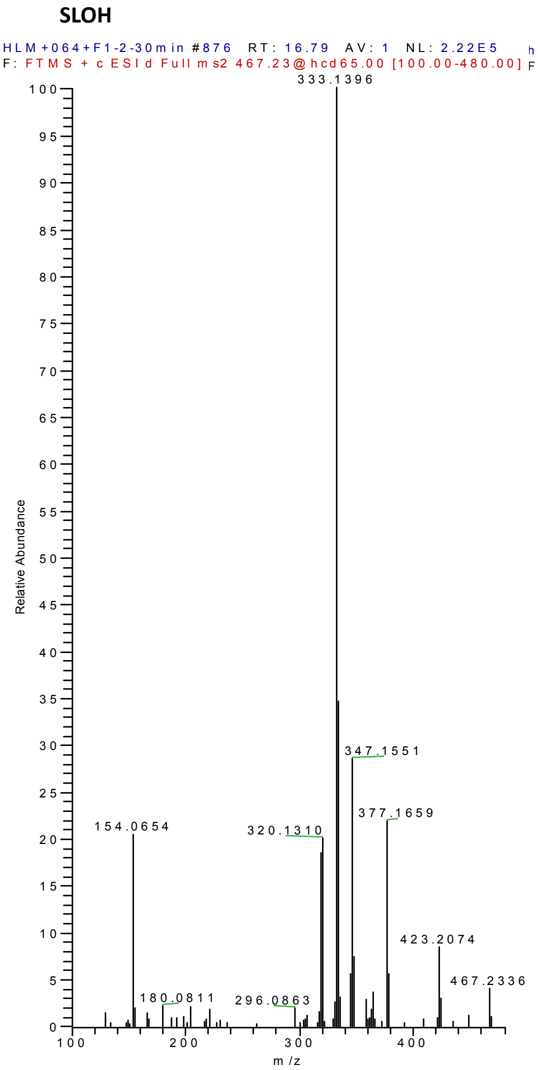
SLOH
HLM+064+F1-2-30min #876 RT: 16.79 AV: 1 NL: 2.22E5
F: FTMS + c ESI d Full ms2 467.23@hcd65.00 [100.00-480.00]
333.1396
347.1551
377.1659
154.0654
320.1310
423.2074
467.2336
180.0811
296.0863
Relative Abundance
m/z

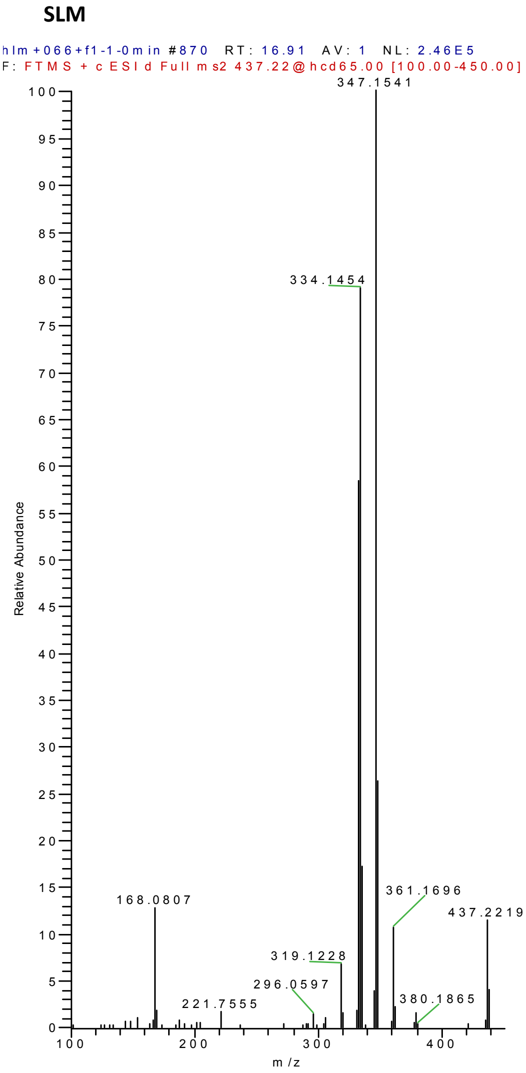
SLM
hlm+066+f1-1-0min #870 RT: 16.91 AV: 1 NL: 2.46E5
F: FTMS + c ESI d Full ms2 437.22@hcd65.00 [100.00-450.00]
347.1541
334.1454
361.1696
437.2219
168.0807
319.1228
296.0597
221.7555
380.1865
Relative Abundance
m/z

Fig. S2

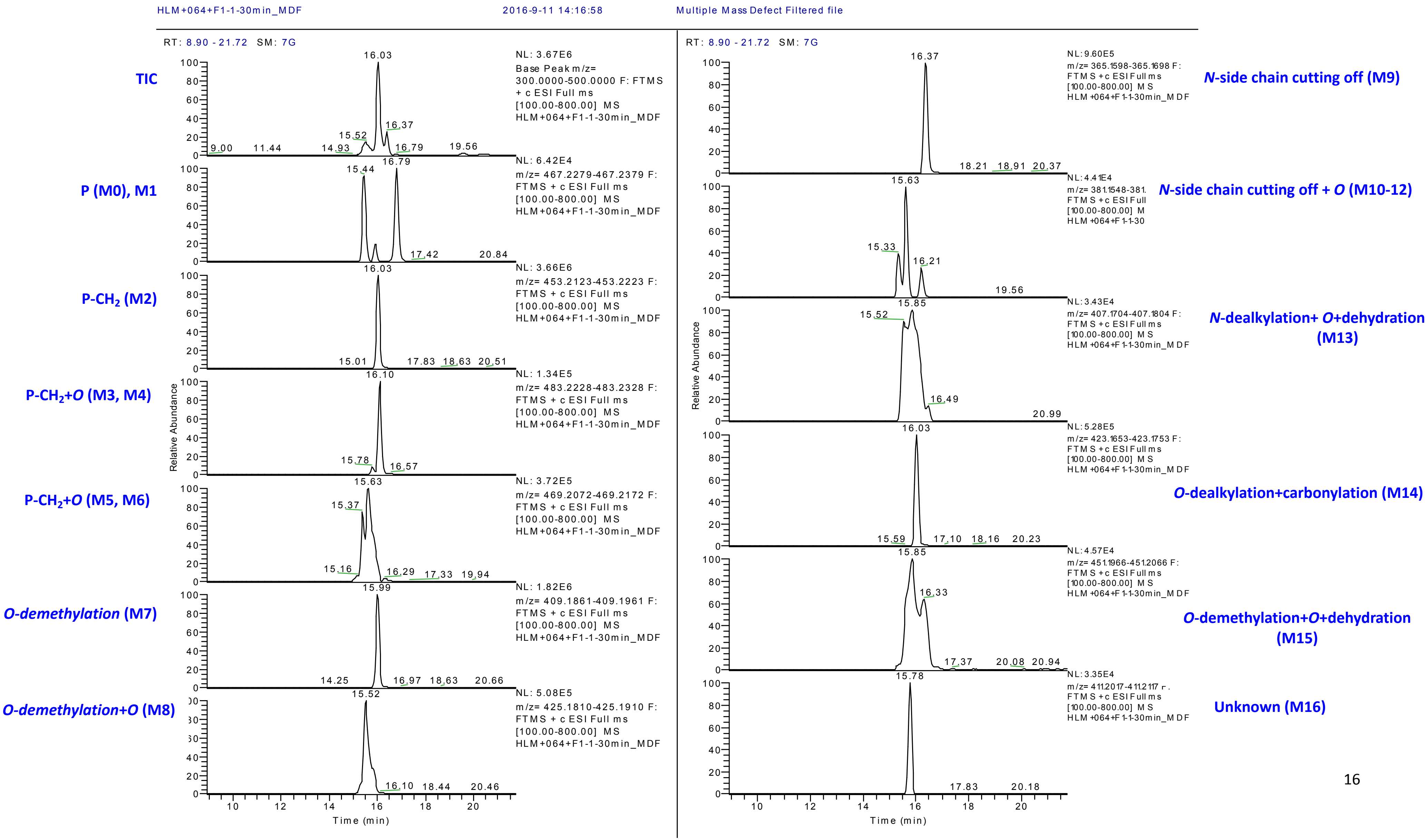
HLM+064+F1-1-30min_MDF
2016-9-11 14:16:58
Multiple Mass Defect Filtered file
RT: 8.90 - 21.72 SM: 7G
TIC
NL: 3.67E6
Base Peak m/z= 300.0000-500.0000 F: FTMS + c ESI Full ms [100.00-800.00] MS HLM+064+F1-1-30min_MDF
P (M0), M1
NL: 6.42E4
m/z= 467.2279-467.2379 F: FTMS + c ESI Full ms [100.00-800.00] MS HLM+064+F1-1-30min_MDF
P-CH2 (M2)
NL: 3.66E6
m/z= 453.2123-453.2223 F: FTMS + c ESI Full ms [100.00-800.00] MS HLM+064+F1-1-30min_MDF
P-CH2+O (M3, M4)
NL: 1.34E5
m/z= 483.2228-483.2328 F: FTMS + c ESI Full ms [100.00-800.00] MS HLM+064+F1-1-30min_MDF
P-CH2+O (M5, M6)
NL: 3.72E5
m/z= 469.2072-469.2172 F: FTMS + c ESI Full ms [100.00-800.00] MS HLM+064+F1-1-30min_MDF
O-demethylation (M7)
NL: 1.82E6
m/z= 409.1861-409.1961 F: FTMS + c ESI Full ms [100.00-800.00] MS HLM+064+F1-1-30min_MDF
O-demethylation+O (M8)
NL: 5.08E5
m/z= 425.1810-425.1910 F: FTMS + c ESI Full ms [100.00-800.00] MS HLM+064+F1-1-30min_MDF
Relative Abundance
Time (min)
RT: 8.90 - 21.72 SM: 7G
N-side chain cutting off (M9)
NL: 9.60E5
m/z= 365.1598-365.1698 F: FTMS + c ESI Full ms [100.00-800.00] MS HLM+064+F1-1-30min_MDF
N-side chain cutting off + O (M10-12)
NL: 4.41E4
m/z= 381.1548-381. FTMS + c ESI Full [100.00-800.00] M HLM+064+F1-1-30
N-dealkylation+ O+dehydration (M13)
NL: 3.43E4
m/z= 407.1704-407.1804 F: FTMS + c ESI Full ms [100.00-800.00] MS HLM+064+F1-1-30min_MDF
O-dealkylation+carbonylation (M14)
NL: 5.28E5
m/z= 423.1653-423.1753 F: FTMS + c ESI Full ms [100.00-800.00] MS HLM+064+F1-1-30min_MDF
O-demethylation+O+dehydration (M15)
NL: 4.57E4
m/z= 451.1966-451.2066 F: FTMS + c ESI Full ms [100.00-800.00] MS HLM+064+F1-1-30min_MDF
Unknown (M16)
NL: 3.35E4
m/z= 411.2017-411.2117 F: FTMS + c ESI Full ms [100.00-800.00] MS HLM+064+F1-1-30min_MDF
Relative Abundance
Time (min)

Fig. S3

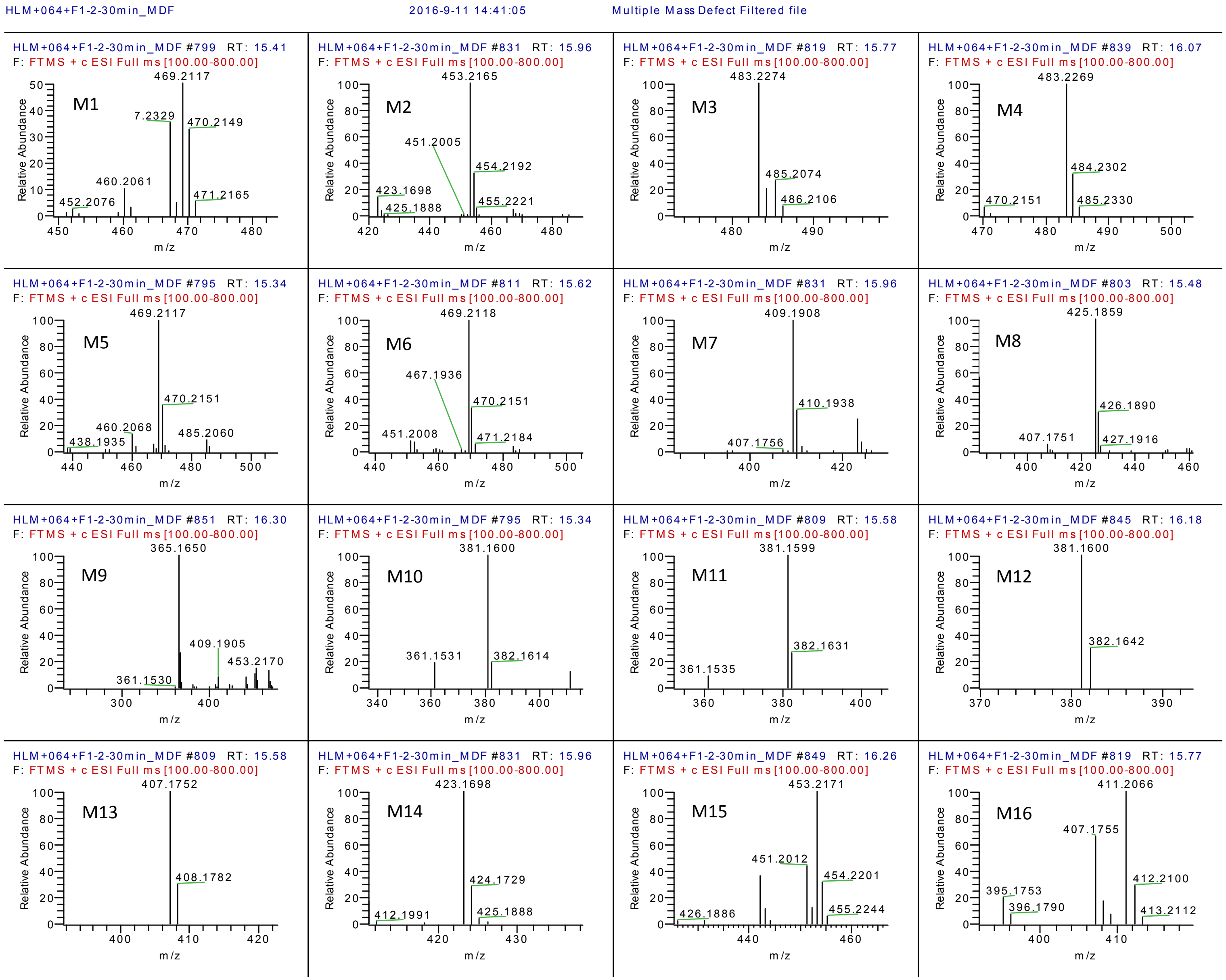

HLM+064+F1-2-30min_MDF
2016-9-11 14:41:05
Multiple Mass Defect Filtered file
HLM+064+F1-2-30min_MDF #799 RT: 15.41
F: FTMS + c ESI Full ms [100.00-800.00]
M1
469.2117
7.2329
470.2149
460.2061
452.2076
471.2165
HLM+064+F1-2-30min_MDF #831 RT: 15.96
F: FTMS + c ESI Full ms [100.00-800.00]
M2
453.2165
451.2005
454.2192
423.1698
425.1888
455.2221
HLM+064+F1-2-30min_MDF #819 RT: 15.77
F: FTMS + c ESI Full ms [100.00-800.00]
M3
483.2274
485.2074
486.2106
HLM+064+F1-2-30min_MDF #839 RT: 16.07
F: FTMS + c ESI Full ms [100.00-800.00]
M4
483.2269
484.2302
470.2151
485.2330
HLM+064+F1-2-30min_MDF #795 RT: 15.34
F: FTMS + c ESI Full ms [100.00-800.00]
M5
469.2117
470.2151
460.2068
438.1935
485.2060
HLM+064+F1-2-30min_MDF #811 RT: 15.62
F: FTMS + c ESI Full ms [100.00-800.00]
M6
469.2118
467.1936
470.2151
451.2008
471.2184
HLM+064+F1-2-30min_MDF #831 RT: 15.96
F: FTMS + c ESI Full ms [100.00-800.00]
M7
409.1908
410.1938
407.1756
HLM+064+F1-2-30min_MDF #803 RT: 15.48
F: FTMS + c ESI Full ms [100.00-800.00]
M8
425.1859
426.1890
407.1751
427.1916
HLM+064+F1-2-30min_MDF #851 RT: 16.30
F: FTMS + c ESI Full ms [100.00-800.00]
M9
365.1650
409.1905
453.2170
361.1530
HLM+064+F1-2-30min_MDF #795 RT: 15.34
F: FTMS + c ESI Full ms [100.00-800.00]
M10
381.1600
361.1531
382.1614
HLM+064+F1-2-30min_MDF #809 RT: 15.58
F: FTMS + c ESI Full ms [100.00-800.00]
M11
381.1599
382.1631
361.1535
HLM+064+F1-2-30min_MDF #845 RT: 16.18
F: FTMS + c ESI Full ms [100.00-800.00]
M12
381.1600
382.1642
HLM+064+F1-2-30min_MDF #809 RT: 15.58
F: FTMS + c ESI Full ms [100.00-800.00]
M13
407.1752
408.1782
HLM+064+F1-2-30min_MDF #831 RT: 15.96
F: FTMS + c ESI Full ms [100.00-800.00]
M14
423.1698
424.1729
412.1991
425.1888
HLM+064+F1-2-30min_MDF #849 RT: 16.26
F: FTMS + c ESI Full ms [100.00-800.00]
M15
453.2171
451.2012
454.2201
426.1886
455.2244
HLM+064+F1-2-30min_MDF #819 RT: 15.77
F: FTMS + c ESI Full ms [100.00-800.00]
M16
411.2066
407.1755
395.1753
396.1790
412.2100
413.2112
Relative Abundance
m/z

Fig. S4

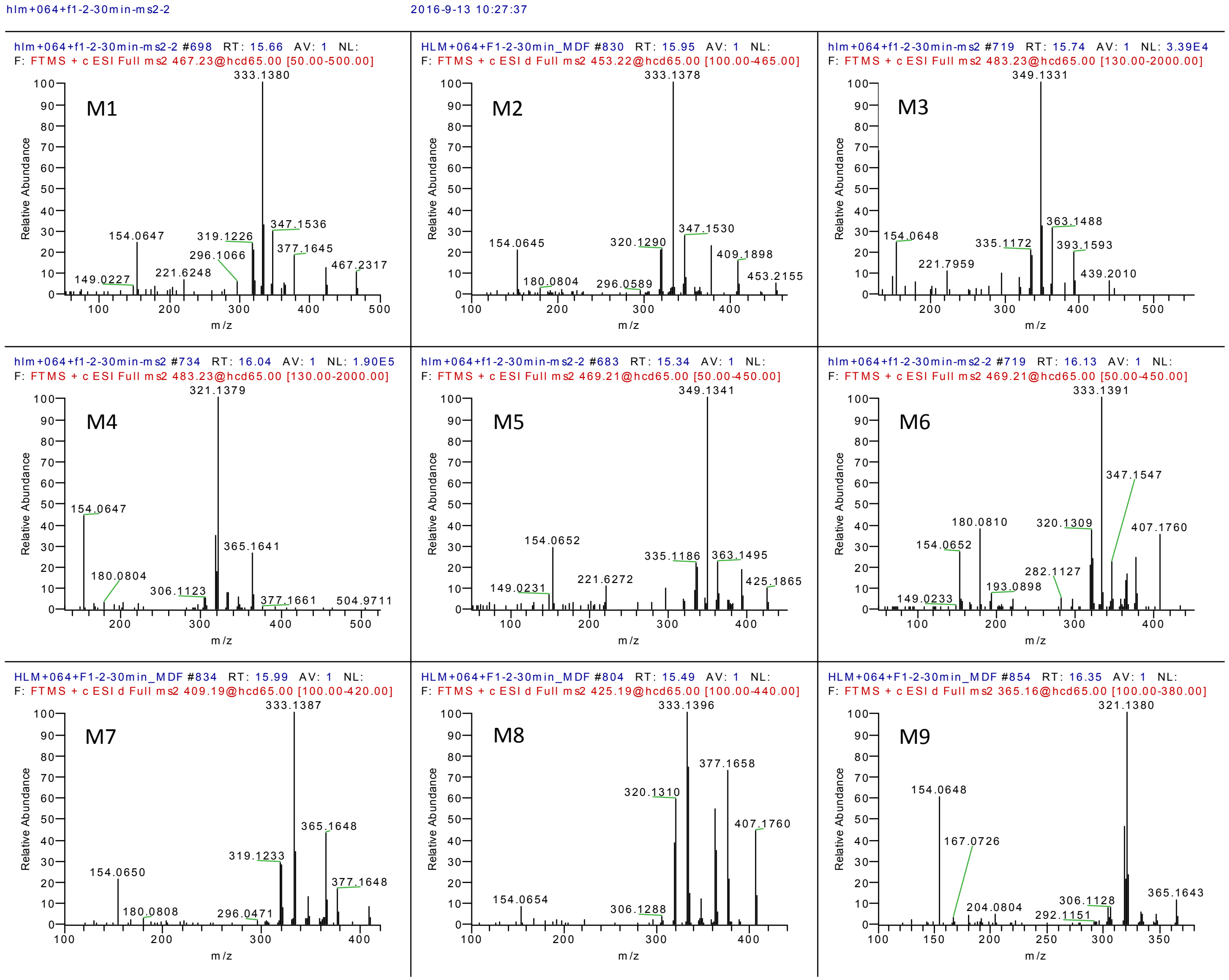

Continued

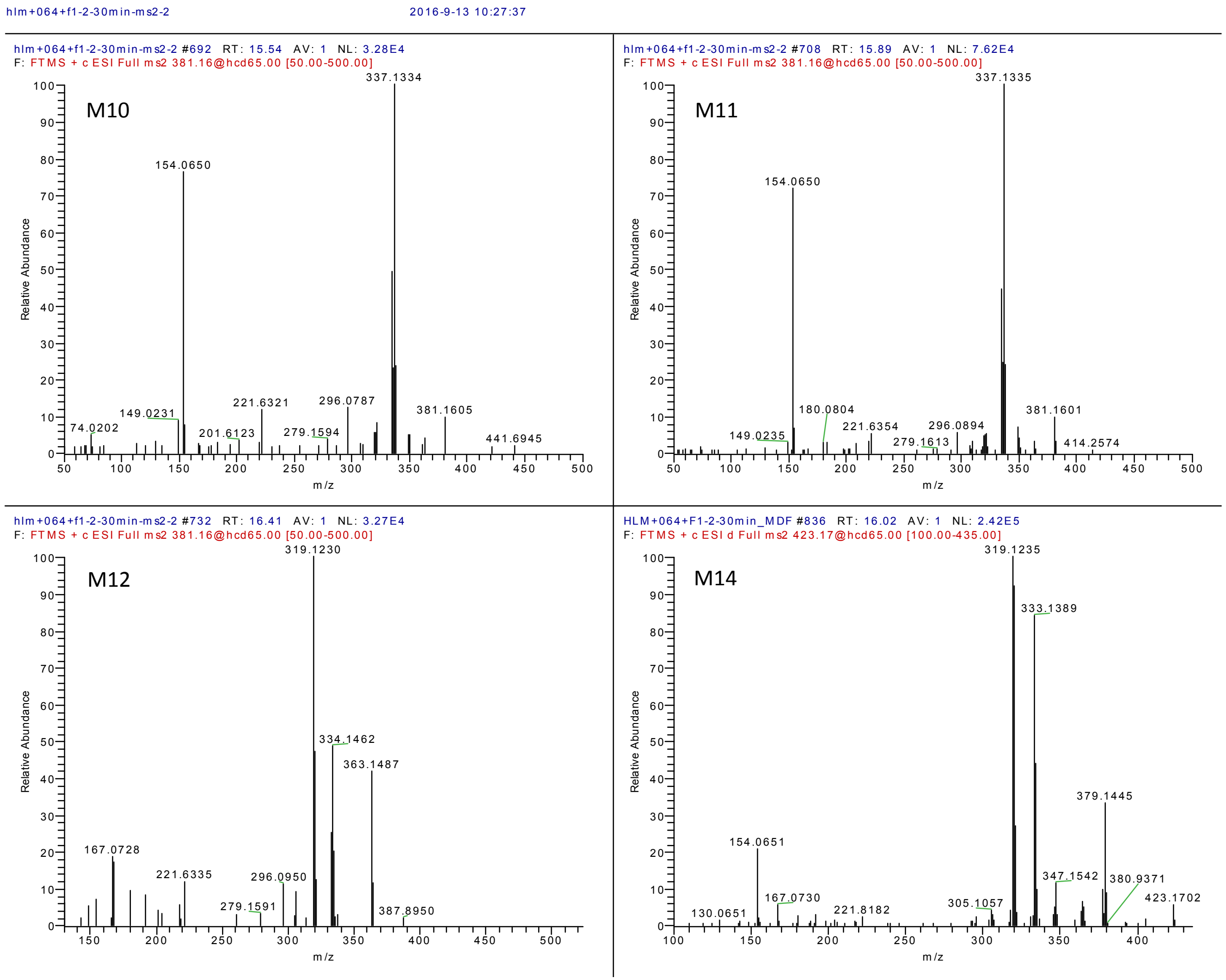
hlm+064+f1-2-30min-ms2-2
2016-9-13 10:27:37
hlm+064+f1-2-30min-ms2-2 #692 RT: 15.54 AV: 1 NL: 3.28E4
F: FTMS + c ESI Full ms2 381.16@hcd65.00 [50.00-500.00]
M10
337.1334
154.0650
74.0202
149.0231
201.6123
221.6321
279.1594
296.0787
381.1605
441.6945
hlm+064+f1-2-30min-ms2-2 #708 RT: 15.89 AV: 1 NL: 7.62E4
F: FTMS + c ESI Full ms2 381.16@hcd65.00 [50.00-500.00]
M11
337.1335
154.0650
149.0235
180.0804
221.6354
279.1613
296.0894
381.1601
414.2574
hlm+064+f1-2-30min-ms2-2 #732 RT: 16.41 AV: 1 NL: 3.27E4
F: FTMS + c ESI Full ms2 381.16@hcd65.00 [50.00-500.00]
M12
319.1230
334.1462
363.1487
167.0728
221.6335
279.1591
296.0950
387.8950
HLM+064+F1-2-30min_MDF #836 RT: 16.02 AV: 1 NL: 2.42E5
F: FTMS + c ESI d Full ms2 423.17@hcd65.00 [100.00-435.00]
M14
319.1235
333.1389
379.1445
154.0651
130.0651
167.0730
221.8182
305.1057
347.1542
380.9371
423.1702
Relative Abundance
m/z

Fig. S5

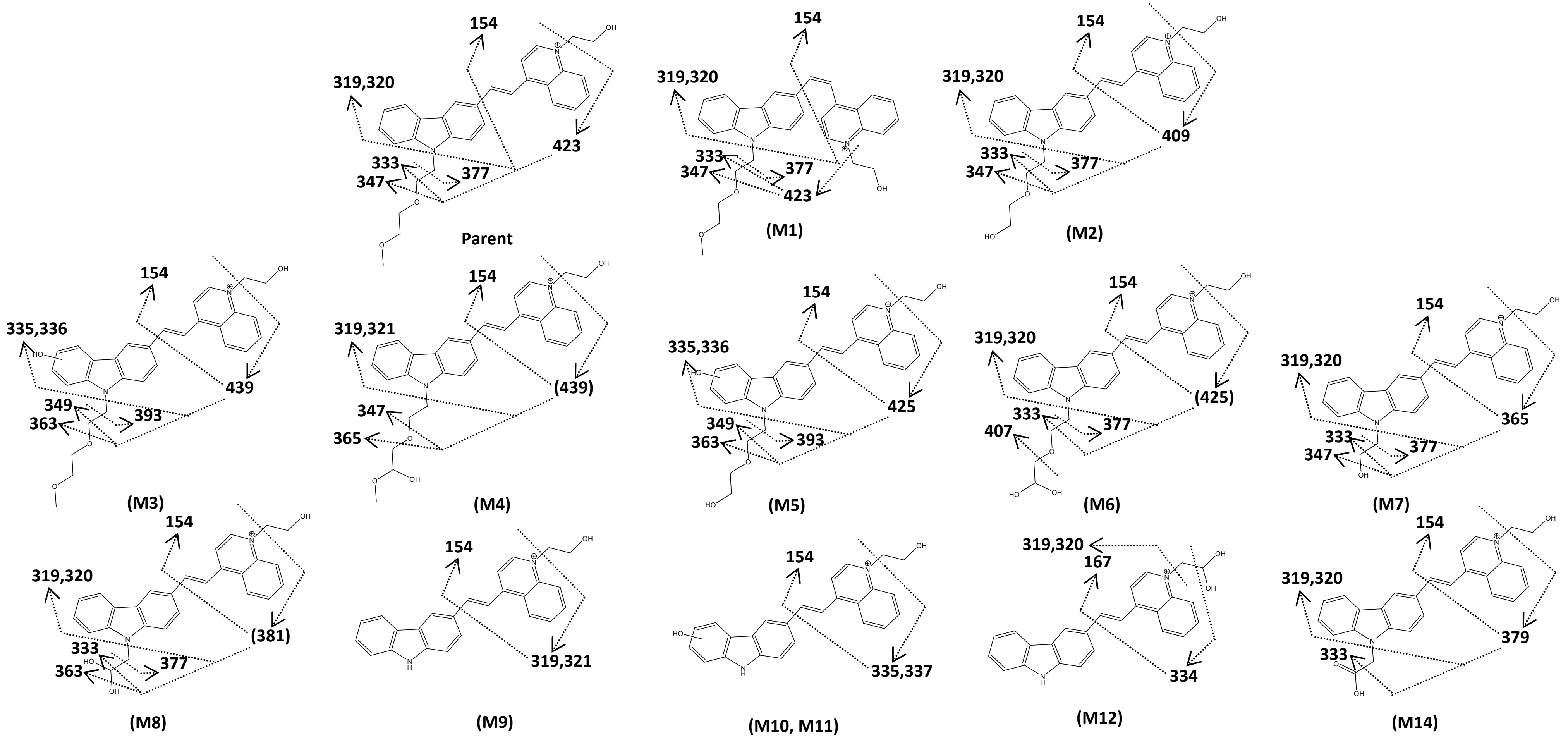
154
319,320
423
333
347
377
Parent
154
319,320
333
347
377
423
(M1)
154
319,320
409
333
347
377
(M2)
154
335,336
439
349
363
393
(M3)
154
319,321
(439)
347
365
(M4)
154
335,336
425
349
363
393
(M5)
154
319,320
(425)
333
407
377
(M6)
154
319,320
365
333
347
377
(M7)
154
319,320
(381)
333
363
377
(M8)
154
319,321
(M9)
154
335,337
(M10, M11)
319,320
167
334
(M12)
154
319,320
379
333
(M14)

Fig. S6

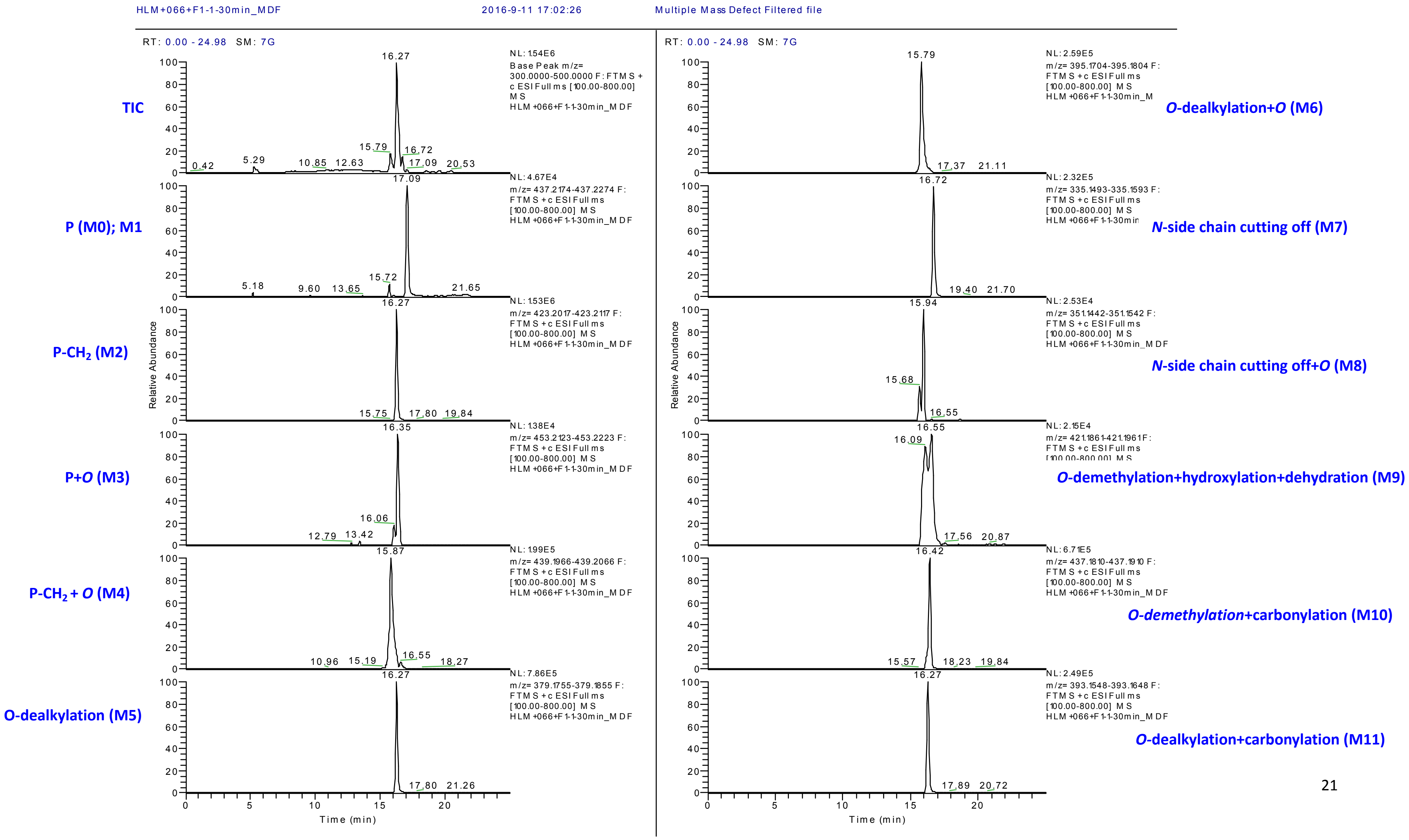
HLM+066+F1-1-30min_MDF
2016-9-11 17:02:26
Multiple Mass Defect Filtered file
RT: 0.00 - 24.98 SM: 7G
TIC
P (M0); M1
P-CH2 (M2)
P+O (M3)
P-CH2 + O (M4)
O-dealkylation (M5)
O-dealkylation+O (M6)
N-side chain cutting off (M7)
N-side chain cutting off+O (M8)
O-demethylation+hydroxylation+dehydration (M9)
O-demethylation+carbonylation (M10)
O-dealkylation+carbonylation (M11)
NL: 1.54E6 Base Peak m/z= 300.0000-500.0000 F: FTMS + c ESI Full ms [100.00-800.00] MS HLM+066+F1-1-30min_MDF
NL: 4.67E4 m/z= 437.2174-437.2274 F: FTMS +c ESI Full ms [100.00-800.00] MS HLM+066+F1-1-30min_MDF
NL: 1.53E6 m/z= 423.2017-423.2117 F: FTMS +c ESI Full ms [100.00-800.00] MS HLM+066+F1-1-30min_MDF
NL: 1.38E4 m/z= 453.2123-453.2223 F: FTMS +c ESI Full ms [100.00-800.00] MS HLM+066+F1-1-30min_MDF
NL: 1.99E5 m/z= 439.1966-439.2066 F: FTMS +c ESI Full ms [100.00-800.00] MS HLM+066+F1-1-30min_MDF
NL: 7.86E5 m/z= 379.1755-379.1855 F: FTMS +c ESI Full ms [100.00-800.00] MS HLM+066+F1-1-30min_MDF
NL: 2.59E5 m/z= 395.1704-395.1804 F: FTMS +c ESI Full ms [100.00-800.00] MS HLM+066+F1-1-30min_M
NL: 2.32E5 m/z= 335.1493-335.1593 F: FTMS +c ESI Full ms [100.00-800.00] MS HLM+066+F1-1-30min
NL: 2.53E4 m/z= 351.1442-351.1542 F: FTMS +c ESI Full ms [100.00-800.00] MS HLM+066+F1-1-30min_MDF
NL: 2.15E4 m/z= 421.1861-421.1961 F: FTMS +c ESI Full ms
NL: 6.71E5 m/z= 437.1810-437.1910 F: FTMS +c ESI Full ms [100.00-800.00] MS HLM+066+F1-1-30min_MDF
NL: 2.49E5 m/z= 393.1548-393.1648 F: FTMS +c ESI Full ms [100.00-800.00] MS HLM+066+F1-1-30min_MDF
Relative Abundance
Time (min)

Fig. S7

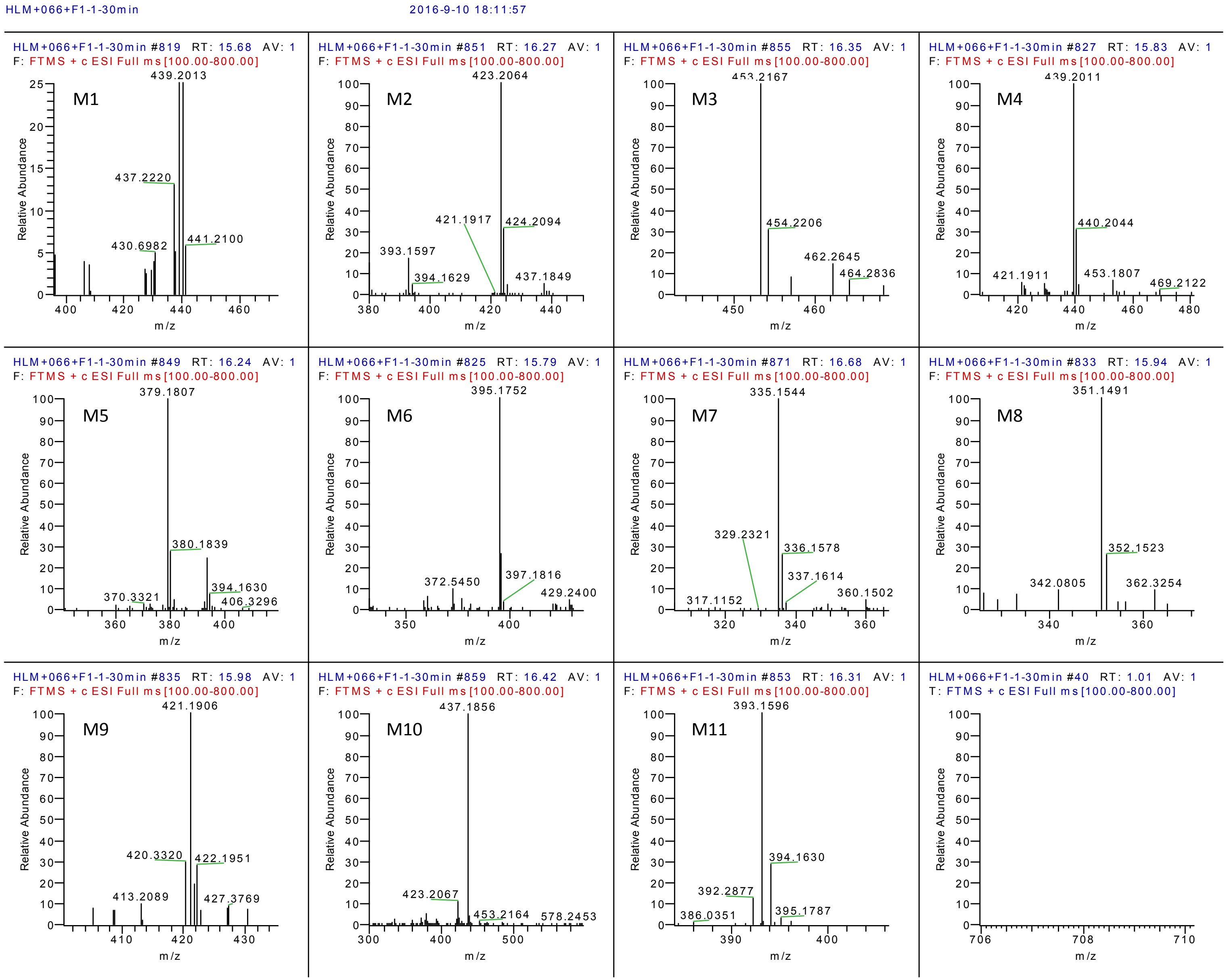
HLM+066+F1-1-30min
2016-9-10 18:11:57
HLM+066+F1-1-30min #819 RT: 15.68 AV: 1
F: FTMS + c ESI Full ms [100.00-800.00]
M1
439.2013
437.2220
430.6982
441.2100
HLM+066+F1-1-30min #851 RT: 16.27 AV: 1
F: FTMS + c ESI Full ms [100.00-800.00]
M2
423.2064
421.1917
424.2094
393.1597
394.1629
437.1849
HLM+066+F1-1-30min #855 RT: 16.35 AV: 1
F: FTMS + c ESI Full ms [100.00-800.00]
M3
453.2167
454.2206
462.2645
464.2836
HLM+066+F1-1-30min #827 RT: 15.83 AV: 1
F: FTMS + c ESI Full ms [100.00-800.00]
M4
439.2011
440.2044
421.1911
453.1807
469.2122
HLM+066+F1-1-30min #849 RT: 16.24 AV: 1
F: FTMS + c ESI Full ms [100.00-800.00]
M5
379.1807
380.1839
370.3321
394.1630
406.3296
HLM+066+F1-1-30min #825 RT: 15.79 AV: 1
F: FTMS + c ESI Full ms [100.00-800.00]
M6
395.1752
372.5450
397.1816
429.2400
HLM+066+F1-1-30min #871 RT: 16.68 AV: 1
F: FTMS + c ESI Full ms [100.00-800.00]
M7
335.1544
329.2321
336.1578
337.1614
317.1152
360.1502
HLM+066+F1-1-30min #833 RT: 15.94 AV: 1
F: FTMS + c ESI Full ms [100.00-800.00]
M8
351.1491
352.1523
342.0805
362.3254
HLM+066+F1-1-30min #835 RT: 15.98 AV: 1
F: FTMS + c ESI Full ms [100.00-800.00]
M9
421.1906
420.3320
422.1951
413.2089
427.3769
HLM+066+F1-1-30min #859 RT: 16.42 AV: 1
F: FTMS + c ESI Full ms [100.00-800.00]
M10
437.1856
423.2067
453.2164
578.2453
HLM+066+F1-1-30min #853 RT: 16.31 AV: 1
F: FTMS + c ESI Full ms [100.00-800.00]
M11
393.1596
394.1630
392.2877
395.1787
386.0351
HLM+066+F1-1-30min #40 RT: 1.01 AV: 1
T: FTMS + c ESI Full ms [100.00-800.00]
Relative Abundance
m/z

Fig. S8

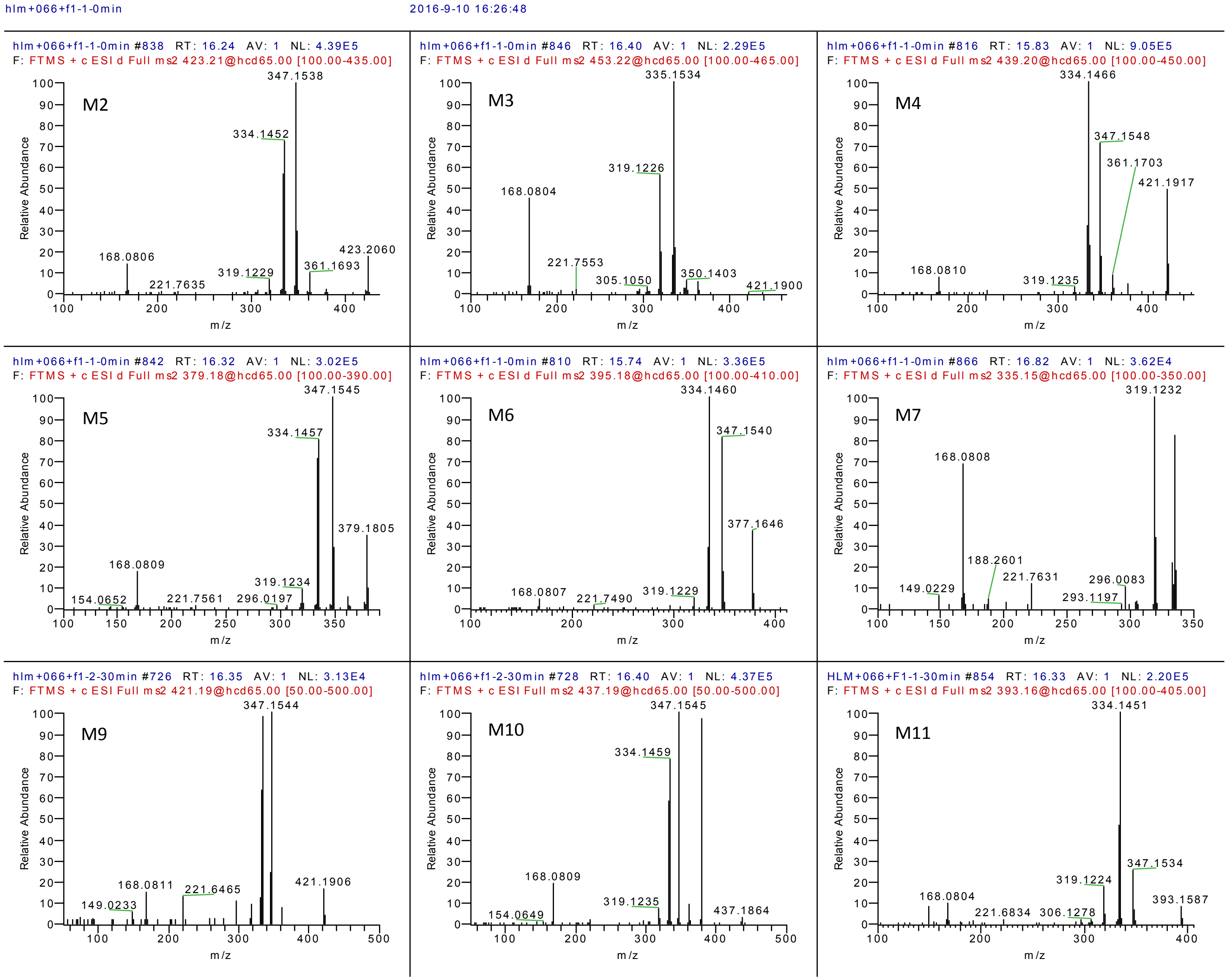
hlm+066+f1-1-0min
2016-9-10 16:26:48
hlm+066+f1-1-0min #838 RT: 16.24 AV: 1 NL: 4.39E5
F: FTMS + c ESI d Full ms2 423.21@hcd65.00 [100.00-435.00]
M2
347.1538
334.1452
168.0806
221.7635
319.1229
361.1693
423.2060
hlm+066+f1-1-0min #846 RT: 16.40 AV: 1 NL: 2.29E5
F: FTMS + c ESI d Full ms2 453.22@hcd65.00 [100.00-465.00]
M3
335.1534
319.1226
168.0804
221.7553
305.1050
350.1403
421.1900
hlm+066+f1-1-0min #816 RT: 15.83 AV: 1 NL: 9.05E5
F: FTMS + c ESI d Full ms2 439.20@hcd65.00 [100.00-450.00]
M4
334.1466
347.1548
361.1703
421.1917
168.0810
319.1235
hlm+066+f1-1-0min #842 RT: 16.32 AV: 1 NL: 3.02E5
F: FTMS + c ESI d Full ms2 379.18@hcd65.00 [100.00-390.00]
M5
347.1545
334.1457
379.1805
168.0809
319.1234
154.0652
221.7561
296.0197
hlm+066+f1-1-0min #810 RT: 15.74 AV: 1 NL: 3.36E5
F: FTMS + c ESI d Full ms2 395.18@hcd65.00 [100.00-410.00]
M6
334.1460
347.1540
377.1646
168.0807
221.7490
319.1229
hlm+066+f1-1-0min #866 RT: 16.82 AV: 1 NL: 3.62E4
F: FTMS + c ESI d Full ms2 335.15@hcd65.00 [100.00-350.00]
M7
319.1232
168.0808
188.2601
221.7631
296.0083
149.0229
293.1197
hlm+066+f1-2-30min #726 RT: 16.35 AV: 1 NL: 3.13E4
F: FTMS + c ESI Full ms2 421.19@hcd65.00 [50.00-500.00]
M9
347.1544
421.1906
168.0811
221.6465
149.0233
hlm+066+f1-2-30min #728 RT: 16.40 AV: 1 NL: 4.37E5
F: FTMS + c ESI Full ms2 437.19@hcd65.00 [50.00-500.00]
M10
347.1545
334.1459
168.0809
319.1235
154.0649
437.1864
HLM+066+F1-1-30min #854 RT: 16.33 AV: 1 NL: 2.20E5
F: FTMS + c ESI d Full ms2 393.16@hcd65.00 [100.00-405.00]
M11
334.1451
347.1534
319.1224
168.0804
393.1587
221.6834
306.1278
Relative Abundance
m/z

Fig. S9

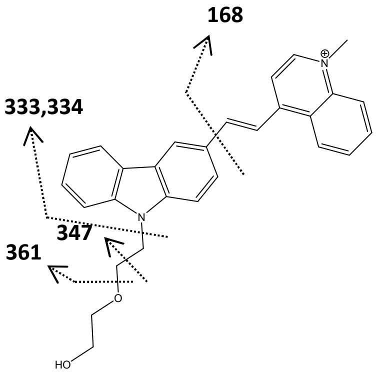

(M2)

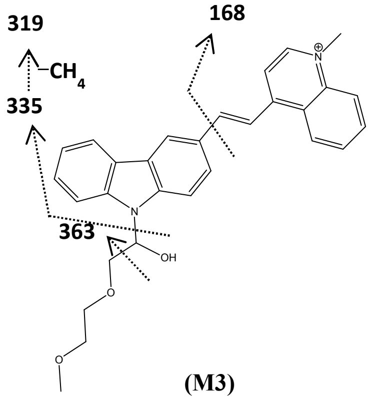

(M3)

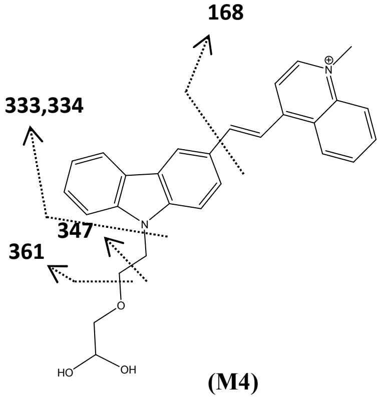

(M4)

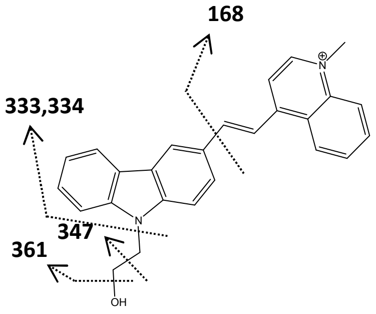

(M5)

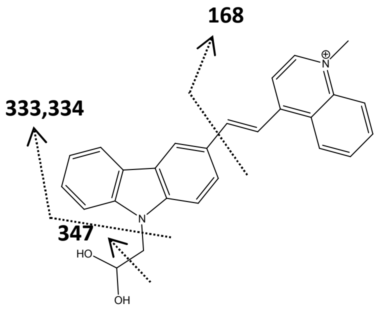

(M6)

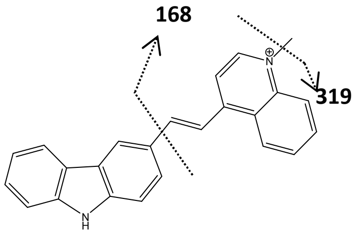

(M7)

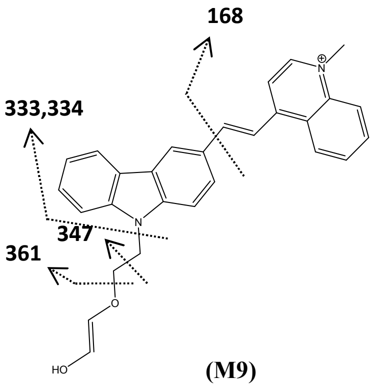

(M9)

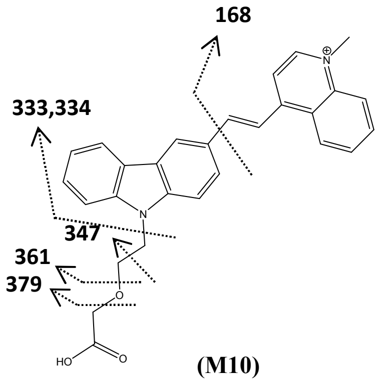

(M10)

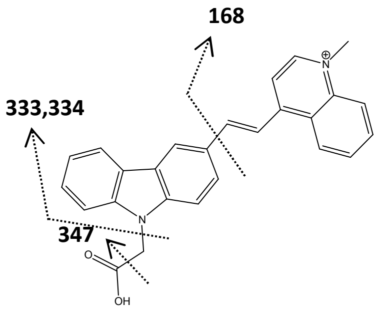

(M11)

Fig. S10

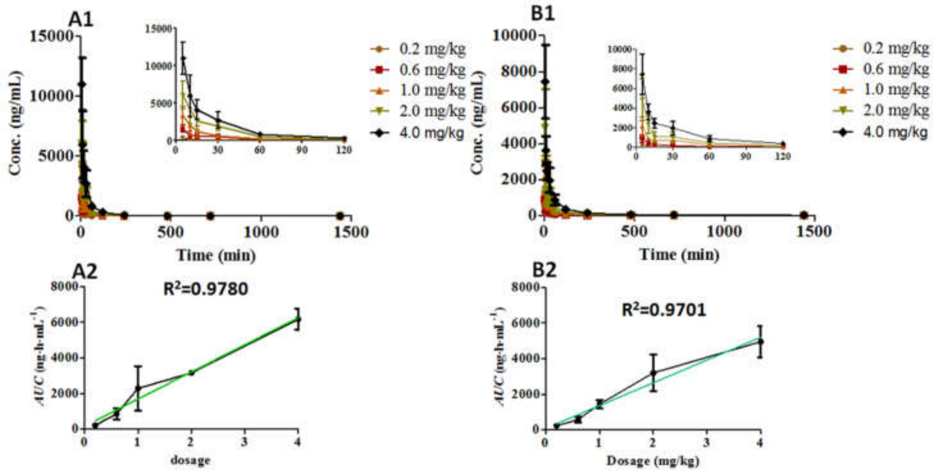

A1
B1
Conc. (ng/mL)
Time (min)
0.2 mg/kg
0.6 mg/kg
1.0 mg/kg
2.0 mg/kg
4.0 mg/kg
A2
B2
R²=0.9780
R²=0.9701
AUC (ng·h·mL⁻¹)
dosage
Dosage (mg/kg)

Fig. S11

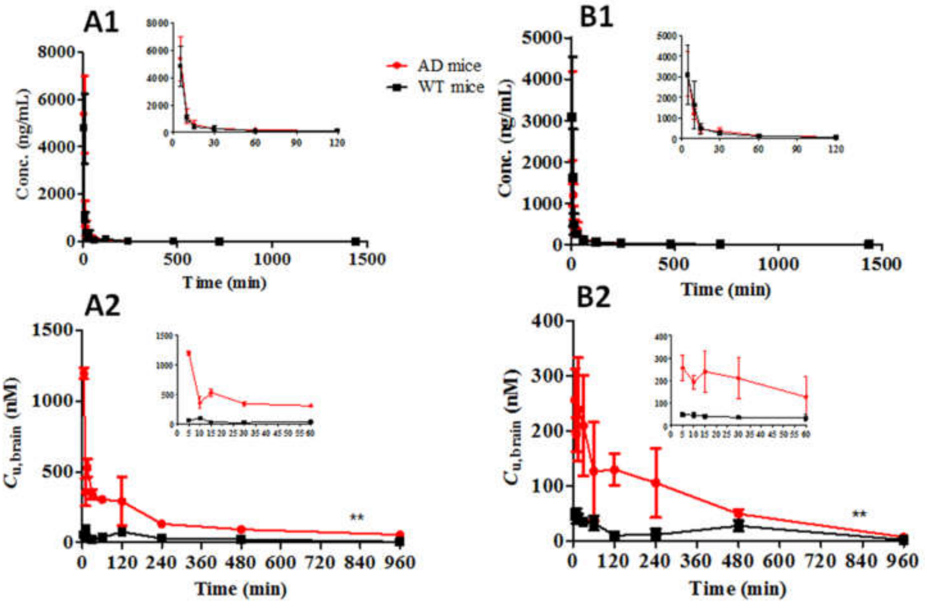

Fig. S12

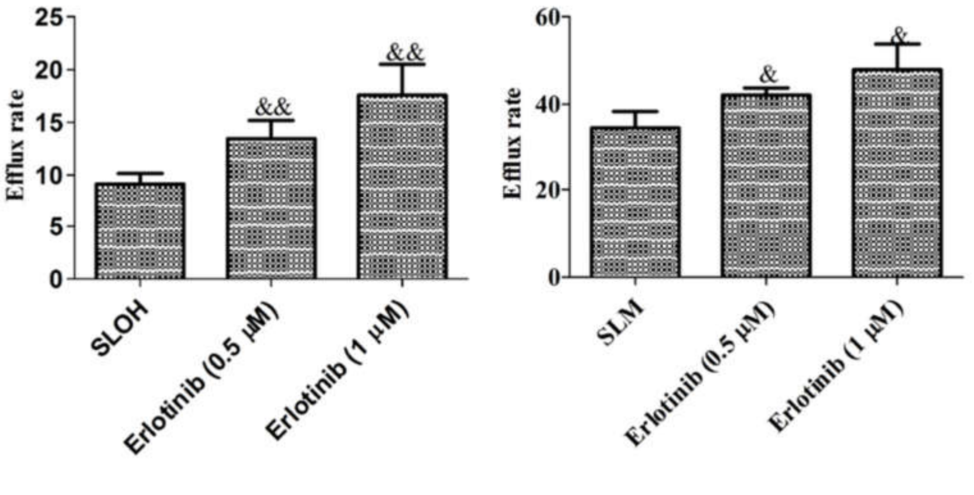

Efflux rate
25
20
15
10
5
0
&&
&&
SLOH
Erlotinib (0.5 μM)
Erlotinib (1 μM)
Efflux rate
60
40
20
0
&
&
SLM
Erlotinib (0.5 μM)
Erlotinib (1 μM)

Fig. S13

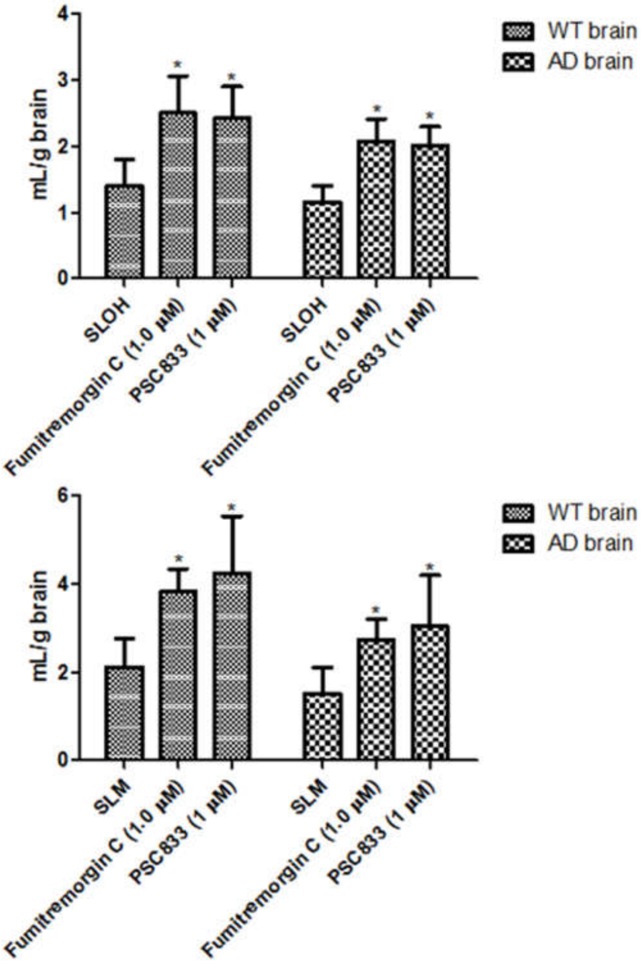
mL/g brain
WT brain
AD brain
SLOH
Fumitremorgin C (1.0 μM)
PSC833 (1 μM)
SLM
